# Korean lunar navigation constellation with stability-filtered optimisation

**DOI:** 10.1038/s41598-026-60190-w

**Published:** 2026-07-08

**Authors:** Seungsoo Yoo, Gyu-In Jee, Sun Yong Kim

**Affiliations:** Department of Electrical and Electronics Engineering, Konkuk University, Seoul, 05029 Republic of Korea

**Keywords:** Lunar navigation, PNT constellation design, Elliptical lunar frozen orbit, Orbital stability filter, Greedy optimisation, KASA 2032 lunar lander, Engineering, Mathematics and computing

## Abstract

The international lunar navigation baseline—eight satellites in elliptical lunar frozen orbits (ELFOs) with $\omega =90^{\circ }$—provides robust south-polar PNT coverage but leaves near-side mid-latitudes largely unserved. This gap directly affects the Korea Aerospace Administration (KASA) 2032 lander mission, whose candidate sites span latitudes $40^{\circ }$–$70^{\circ }$. We propose the Korean Lunar Navigation System (KLNS): five augmentation satellites in $\omega =270^{\circ }$ ELFOs that concentrate dwell time over the northern hemisphere. A two-stage optimisation is employed. First, every candidate orbit in a 10,368-element search space is propagated for 720 hours under a perturbed model (lunar $J_2$ + Earth third-body) and screened against four stability criteria, removing 2864 (27.6%) dynamically infeasible candidates. Second, a greedy sequential algorithm with hard S-ROI protection selects five KLNS satellites that raise N40–70 PNT availability from 0.0 to 89.5%, far exceeding the 50% target, while preserving 99.7% south-polar coverage. The stability-filtered greedy solution outperforms a *100× 50* genetic-algorithm benchmark (85.6%), demonstrating that a well-designed pre-filter can obviate more expensive metaheuristics. Site-specific evaluation confirms 92.5–94.2% availability at all three northern KASA candidate landing sites under the augmented constellation.

## Introduction

Terrestrial users are served by global navigation satellite systems (GNSS) such as GPS and Galileo, but these signals are poorly suited to the Moon: GNSS satellites direct their main antenna beams toward the Earth, so a receiver in cislunar space detects only the weak signals that spill past the Earth’s limb, under unfavourable viewing geometry. Reliable lunar operations therefore require dedicated positioning, navigation, and timing (PNT) infrastructure placed in orbit around the Moon itself.

As multiple space agencies prepare for sustained lunar surface operations, the need for reliable cislunar PNT infrastructure has become a central challenge. NASA’s LunaNet architecture^1^, whose interoperability specification (LNIS) reached Version 5 baseline in early 2025^2^, ESA’s Moonlight programme^3^, and JAXA’s contributions^4^ collectively envision an eight-satellite constellation in elliptical lunar frozen orbits (ELFOs) with argument of periapsis $\omega =90^{\circ }$—the argument of periapsis *ω* sets the orientation of the elongated orbit, and hence which hemisphere lies beneath the slow, high-altitude apoapsis arc where each satellite spends most of its time. This baseline provides outstanding coverage at the lunar south pole—a primary target for the Artemis programme—but concentrates apoapsis dwell over the southern hemisphere, leaving near-side mid-latitudes largely uncovered^5,6^. China has independently demonstrated lunar relay infrastructure through the Queqiao-2 satellite in an elliptical frozen orbit, providing far-side and south-pole communication relay since 2024^7^. Meanwhile, the Firefly Blue Ghost lander achieved the first GPS/GNSS signal lock on the lunar surface in 2025, demonstrating potential for Earth-based GNSS augmentation of lunar PNT^8^ and validating earlier theoretical analyses of lunar GNSS receiver sensitivity and acquisition^9^.

The Korea Aerospace Administration (KASA) is developing a lunar lander for launch in 2032, with candidate landing sites in the latitude range $40^{\circ }$–$70^{\circ }$ (both hemispheres). On 25 March 2026, KASA held a public hearing at the Korea Institute of Geoscience and Mineral Resources (KIGAM) and formally announced six representative candidate landing sites within the $40^{\circ }$–$70^{\circ }$ latitude band: three in the northern hemisphere (Gärtner Crater at $59.24^{\circ }\mathrm{N}$, Endymion Crater at $53.61^{\circ }\mathrm{N}$, and Lacus Mortis at $45.13^{\circ }\mathrm{N}$) and three in the southern hemisphere (Clavius Crater at $58.62^{\circ }\mathrm{S}$, Pingré Crater at $58.64^{\circ }\mathrm{S}$, and Maginus Crater at $50.03^{\circ }\mathrm{S}$)^10,11^. These sites were selected based on scientific exploration value, terrain stability, and the ability to sustain surface operations for at least ten days. A final selection is planned by the end of 2026.

This paper addresses the PNT coverage gap by designing the Korean Lunar Navigation System (KLNS)—a five-satellite augmentation constellation specifically tailored to serve the KASA candidate sites. The key innovations are twofold. First, the use of $\omega =270^{\circ }$ ELFOs places apoapsis over the northern hemisphere, providing complementary dwell characteristics to the baseline $\omega =90^{\circ }$ constellation^12,13^. Second, and central to this work, the search space is screened by a 720-h orbital stability pre-filter *before* the optimiser runs. Stability is enforced through four quantitative criteria (eccentricity bound, semi-major axis variation, inclination drift, and minimum periapsis), which together remove 2864 candidate orbits that experience orbital collapse, hyperbolic transition, or excessive inclination drift under perturbed dynamics (lunar $J_2$ and Earth third-body). This pre-filter eliminates the orbital instability observed in preliminary studies based on a short-horizon (168-h) optimiser, where high-eccentricity (*e=0.70*), high-altitude ($a=12{,}000$ km) candidates appeared optimal at 7 days but collapsed near *t ≈ 600* h under 30-day propagation^13^.

The constellation is then optimised over the stability-filtered search space using a greedy sequential algorithm with a hard S-ROI protection constraint and duplicate-orbit exclusion. Performance is quantified through PNT availability (percentage of time with *\ge 4* visible satellites), mean number of visible satellites (NVS), and geometric dilution of precision (GDOP), assessed over dual regions of interest (ROIs) spanning latitudes $40^{\circ }$–$70^{\circ }$ in each hemisphere.

The remainder of this section provides the mission and methodological context: the international lunar navigation baseline against which KLNS augments (Section “International lunar navigation baseline”), the KASA 2032 lander mission requirements that motivate this study (Section “KASA 2032 lunar lander mission”), and the prior literature on lunar constellation design (Section “Related work”).

### International lunar navigation baseline

The baseline constellation considered in this study comprises eight satellites operated by three agencies, as summarised in Table 1. NASA contributes three Lunar Communication Relay and Navigation Satellites (LCRNS) and ESA four NAVSATs, all in $\omega =90^{\circ }$ ELFOs with semi-major axes of approximately 9750–11,300 km and eccentricities of 0.63–0.70. JAXA provides one Lunar Navigation Satellite System (LNSS) spacecraft. All eight satellites share $\omega =90^{\circ }$, placing their apoapsis (and hence longest dwell) over the southern hemisphere, which yields excellent south-polar coverage but minimal presence over northern mid-latitudes.

**Table 1:** Baseline international lunar navigation constellation (8 satellites).

Sat.	a (km)	e	i (°)	*Ω* (°)	*ω* (°)	$M_0$ (°)	Agency
LCRNS-1	11,300	0.63	57.0	0.0	90	0.0	NASA
LCRNS-2	11,300	0.63	57.0	120.0	90	55.0	NASA
LCRNS-3	11,300	0.63	57.0	240.0	90	110.0	NASA
NAVSAT-1	9751	0.70	63.2	30.0	90	0.0	ESA
NAVSAT-2	9751	0.70	63.2	120.0	90	90.0	ESA
NAVSAT-3	9751	0.70	63.2	210.0	90	180.0	ESA
NAVSAT-4	9751	0.70	63.2	300.0	90	270.0	ESA
LNSS-1	9750	0.70	63.2	75.0	90	45.0	JAXA

### KASA 2032 lunar lander mission

Korea’s lunar exploration follows a three-phase roadmap^14^: (1) baseline surface and resource exploration, (2) polar geology and resource prospecting, and (3) long-term observation and in-situ resource utilisation (ISRU) toward a 2040s outpost. The 2032 lander, launched aboard the next-generation Korean launch vehicle, represents Phase 1 of the officially launched lunar lander development project^15^, with science objectives spanning surface dust interactions, chemical composition, and terrain/geological characterisation for future bases^10^. At the 25 March 2026 public hearing, KASA defined the landing latitude as $40^{\circ }$–$70^{\circ }$ to ensure *\ge 10*-day surface operations with adequate solar illumination and communication geometry^10,16^, and identified six representative sites (Table 2) with *∼*30 additional candidates under detailed analysis.

**Table 2:** KASA 2032 candidate landing sites announced at the 25 March 2026 public hearing^10^.

#	Site	Lat.	Lon.	Hem.	Primary science value
1	Gärtner crater	$59.24^{\circ }$N	$34.6^{\circ }$E	N	Volcanic layering; mare formation
2	Endymion crater	$53.61^{\circ }$N	$56.5^{\circ }$E	N	Mantle material analysis (4 km depth)
3	Lacus mortis	$45.13^{\circ }$N	$27.2^{\circ }$E	N	Hexagonal lava plain; lava tubes
4	Clavius crater	$58.62^{\circ }$S	$14.4^{\circ }$W	S	Second-largest crater; water molecules
5	Pingré crater	$58.64^{\circ }$S	$73.7^{\circ }$W	S	Polar region connectivity
6	Maginus crater	$50.03^{\circ }$S	$6.2^{\circ }$W	S	Impact history; volcanic traces

The KLNS constellation designed in this study is specifically optimised to provide PNT coverage across the $40^{\circ }$–$70^{\circ }$ latitude band, directly addressing the operational requirements of the KASA 2032 lander at these candidate sites.

### Related work

Lunar navigation constellation design has matured through a sequence of foundational works. Ely and Lieb^12^ demonstrated polar/global coverage by elliptical inclined orbits, and Folta and Quinn^13^ characterised lunar frozen orbits and the Lidov–Kozai constraints on *(e,i,ω )* for long-term stability. Subsequent system studies refined GDOP/visibility analyses for ELFO architectures^4,6^; Arcia Gil et al.^17^ explored Pareto trade-offs via multi-objective evolutionary algorithms; Bhamidipati et al.^18^ designed and compared SmallSat-based lunar navigation and communication satellite system architectures; Wang et al.^31^ investigated multi-orbit constellations combining distant retrograde and Halo orbits as alternatives to ELFO-only designs; and Delépaut et al.^19^ surveyed coverage and visibility limitations of lunar GNSS architectures. A common limitation across these studies is post-hoc stability verification of a 7–14 day optimisation outcome, which can mis-select high-altitude high-eccentricity orbits that collapse beyond 30 days under amplified Earth third-body perturbations growing as $a^3$^20,21^. The present study addresses this gap by integrating long-horizon stability verification *into* the optimisation pipeline as a pre-filter.

On the infrastructure side, the LNIS V5 baselined by NASA, ESA and JAXA defines the Augmented Forward Signal (AFS) for one-way ranging-based PNT^2,22^; China deployed Queqiao-2 in an elliptical frozen orbit in 2024^7^; and ESA’s Moonlight programme has secured commercial interoperability agreements^3^. Korean foundations include the DTN demonstration aboard the Korea Pathfinder Lunar Orbiter (Danuri)^23^ and Korean academic LNSS architecture studies for the lunar south pole^24^. The KLNS design extends this body of work by serving KASA’s announced candidate landing sites with an interoperable five-satellite augmentation.

The main contributions of this paper are as follows:

1. Introduction of a 720-h orbital stability pre-filter as an explicit step in lunar constellation design, with four quantitative criteria that remove dynamically infeasible candidates before optimisation.
2. Design of a five-satellite KLNS constellation using $\omega =270^{\circ }$ ELFOs in the stability-filtered search space, achieving 89.5% N40–70 availability while preserving 99.7% south-polar coverage—a 24.8-percentage-point improvement over the unfiltered baseline at the same satellite count.
3. Demonstration that a stability-filtered greedy algorithm outperforms both random search and a genetic-algorithm benchmark, providing both a near-optimal solution and a phased deployment roadmap (initial *→* full operational capability) without additional metaheuristic overhead.
4. Comprehensive robustness analysis: 720-h frozen-orbit element histories for the selected satellites, elevation-mask sensitivity, 15-configuration weight sensitivity (9.8-percentage-point spread), and multi-threshold GDOP analysis (70.5% availability at the strict $\hbox{GDOP}<15$ threshold).
5. Site-specific PNT evaluation at the six KASA candidate landing sites, demonstrating that all three northern sites achieve 92.5–94.2% availability under the augmented constellation (compared with 0.0% baseline), with all three southern sites retaining 99.7–100% coverage.

The remainder of this paper is organised as follows. The Results section presents the optimised KLNS constellation, its incremental and long-term performance, and a series of robustness analyses. The Discussion interprets these findings, their operational implications, and the limitations of the present model. The Methods section details the dynamical model, search space, 720-h stability pre-filter, and greedy optimisation algorithm.

## Methods

**Fig. 1:**
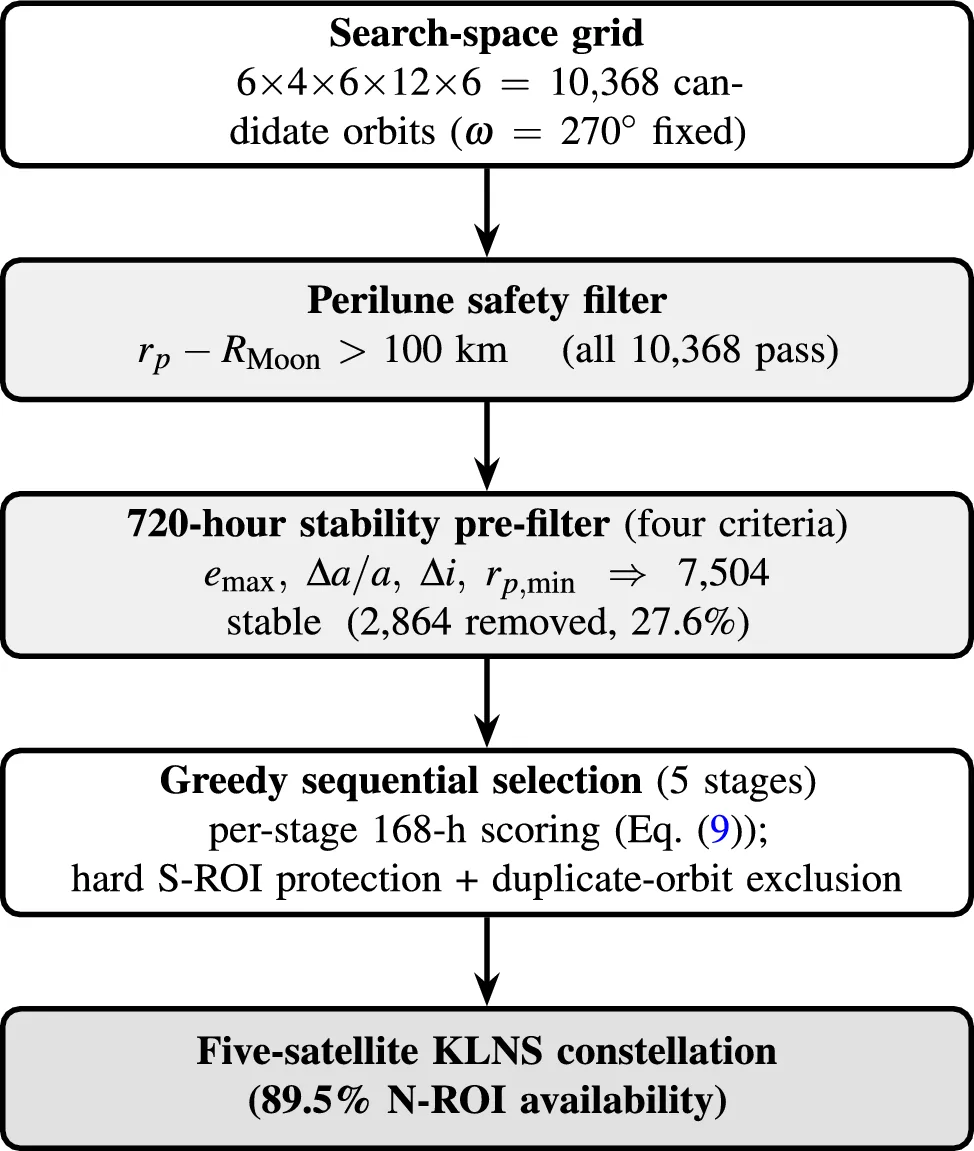
KLNS constellation-design pipeline. An exhaustive orbital grid is reduced by a perilune safety filter and then by the 720-h stability pre-filter (shaded), which removes dynamically infeasible candidates *before* optimisation; a greedy sequential algorithm then selects the five-satellite constellation. The stability pre-filter is the key methodological step that distinguishes this work from post-hoc stability verification.

The optimisation pipeline used in this work consists of four sequential stages: (1) candidate generation, (2) 720-h orbital stability pre-filtering, (3) greedy sequential optimisation under a hard S-ROI protection constraint, and (4) robustness validation including a genetic-algorithm benchmark, elevation-mask sensitivity, weight sensitivity, and site-specific evaluation. The stability pre-filter (described below) is the methodological centrepiece of this paper; without it, the optimiser selects high-altitude, high-eccentricity orbits that appear optimal at the 7-day horizon but collapse over 30 days (Fig. 1).

### Dynamical model

Satellite trajectories are propagated in a Moon-centred inertial reference frame using a fixed-step fourth-order Runge–Kutta (RK4) integrator with a 60-second time step. The instantaneous state vector $\textbf{s} = [\,\textbf{r}^{\textsf{T}}\ \textbf{v}^{\textsf{T}}\,]^{\textsf{T}}$ comprises the position $\textbf{r} = [x,\,y,\,z]^{\textsf{T}}$ and velocity $\textbf{v}$, and evolves according to

1 $$\begin{aligned} \dot{\textbf{r}} = \textbf{v}, \quad \dot{\textbf{v}} = \textbf{a}_{\mathrm{2b}} + \textbf{a}_{J_2} + \textbf{a}_{\mathrm{3b}}, \end{aligned}$$

where $\textbf{a}_{\mathrm{2b}}$, $\textbf{a}_{J_2}$ and $\textbf{a}_{\mathrm{3b}}$ are the lunar central-body (point-mass) attraction, the $J_2$ oblateness perturbation, and the Earth third-body perturbation, respectively.

The central-body acceleration is

2 $$\begin{aligned} \textbf{a}_{\mathrm{2b}} = -\frac{\mu _M}{r^{3}}\,\textbf{r}, \end{aligned}$$

with $r = \Vert \textbf{r} \Vert$ and lunar gravitational parameter $\mu _M = 4902.800066~\hbox{km}^3/\hbox{s}^2$.

The $J_2$ perturbation, capturing the dominant effect of lunar oblateness, is expressed in Cartesian components as

3 $$\begin{aligned} \textbf{a}_{J_2} = -\frac{3}{2}\,\frac{J_2\,\mu _M R_M^{2}}{r^{5}} \begin{bmatrix} \bigl (1 - 5\,(z/r)^{2}\bigr )\,x \\ \bigl (1 - 5\,(z/r)^{2}\bigr )\,y \\ \bigl (3 - 5\,(z/r)^{2}\bigr )\,z \end{bmatrix}, \end{aligned}$$

where $J_2 = 2.033\times 10^{-4}$ is the lunar second zonal harmonic coefficient and $R_M = 1737.4~\mathrm{km}$ is the lunar reference radius.

The Earth third-body perturbation is modelled with a circular approximation for the Earth–Moon system, in which the Earth is held fixed at $\textbf{r}_E = [\,R_{EM},\,0,\,0\,]^{\textsf{T}}$ with mean Earth–Moon distance $R_{EM} = 384{,}400~\mathrm{km}$. The resulting acceleration follows the standard third-body form^20^

4 $$\begin{aligned} \textbf{a}_{\mathrm{3b}} = \mu _E\!\left( \frac{\textbf{r}_E - \textbf{r}}{\Vert \textbf{r}_E - \textbf{r} \Vert ^{3}} - \frac{\textbf{r}_E}{R_{EM}^{3}} \right) , \end{aligned}$$

where $\mu _E = 398{,}600.4418~\hbox{km}^3/\hbox{s}^{2}$ is the Earth gravitational parameter. The first term is the direct attraction of the Earth on the satellite, and the second is the indirect (frame) acceleration arising from the Earth’s attraction on the Moon itself.

Two propagation horizons are used: 168 hours (7 days) for the optimiser score evaluation, and 720 hours (30 days) for both the stability pre-filter and the long-term validation. A fixed step of $\Delta t = 60~\mathrm{s}$ is adopted; a step-size convergence study confirming that this choice is well within the asymptotic regime of the RK4 scheme is reported in Supplementary Table S8. The use of a fixed-step RK4 integrator—rather than an adaptive-step scheme—is standard practice for medium-term Earth- and lunar-orbit propagation at this fidelity, where the smooth, non-impulsive force model produces no rapid transients that would require adaptive step control^28,29^. This $J_2$+third-body model captures the dominant secular and long-period perturbations relevant for ELFO stability assessment^29^. Higher-order lunar gravity harmonics (mascons) and solar radiation pressure are not modelled; their omission is discussed—and quantified against a degree/order-16 GRAIL field in Supplementary Table S9—in the Limitations subsection of the Discussion.

The trajectories are propagated and analysed in the Moon-centred inertial frame. Under the perturbed $J_2$ plus Earth third-body dynamics the selected orbits are *quasi-periodic* rather than strictly periodic: each ground track and element history repeats only approximately, with bounded librations superimposed on the underlying Keplerian motion. The orbital elements reported throughout the paper (and plotted in Fig. 5) are *osculating* classical elements—the instantaneous Keplerian elements of the two-body orbit tangent to the true trajectory at each epoch.

For the selected satellites these osculating elements remain an appropriate and informative representation precisely because the stability pre-filter restricts attention to orbits whose elements oscillate within tight bounds (*Δ a/a < 0.40*, $\Delta i < 10^{\circ }$, $e_{\max } < 0.95$) rather than drifting secularly. In this bounded regime the osculating elements stay close to their mean values, so derived quantities such as periapsis altitude, apoapsis dwell latitude, and inclination retain their usual geometric meaning over the 30-day window—which is what makes the availability and stability assessments physically interpretable. Orbits whose classical elements would instead drift substantially (losing this interpretability and calling for a mean-element or quasi-periodic invariant-torus description) are exactly those that the four stability criteria remove before optimisation. The bounded, quasi-periodic element evolution of every retained satellite (Fig. 5) therefore confirms that the osculating classical-element description is valid for the selected constellation, and that the reported metrics reflect genuinely stable, near-frozen motion rather than artefacts of a drifting coordinate description.

### Performance metrics

Performance is evaluated on a *37 × 36* latitude–longitude grid ($5^{\circ }$ spacing) covering the full Moon, with reporting focused on the near-side hemisphere. At each grid point and each 600-second sample (10-minute output interval), a satellite is deemed visible if its elevation angle exceeds a minimum mask of $\varepsilon _{\min } = 5^{\circ }$ and its line of sight is not blocked by the Moon’s geometric horizon (ray–sphere intersection test). Three metrics are computed:

- **NVS**: mean number of visible satellites, time-averaged over the simulation window.
- **GDOP**: geometric dilution of precision, computed as $\sqrt{\mathrm{tr}((\textbf{H}^\top \textbf{H})^{-1})}$ from the observation matrix $\textbf{H}$ at epochs with NVS *\ge 4*^30^.
- **PNT availability**: percentage of epochs at which NVS *\ge 4* and GDOP < 50. The GDOP < 50 threshold serves as a liberal filter to exclude only geometrically degenerate configurations; the primary availability metric is driven by the NVS *\ge 4* condition. To provide a more nuanced view of navigation quality, we additionally report availability at stricter thresholds (GDOP < 15, < 20, < 30) in the Multi-Threshold GDOP Analysis subsection of Results.

Two regions of interest are defined for the $40^{\circ }$–$70^{\circ }$ latitude band: **N-ROI** (lat $40^{\circ }$–$70^{\circ }$N, lon $-90^{\circ }$–$90^{\circ }$E) and **S-ROI** (lat $40^{\circ }$–$70^{\circ }$S, lon $-90^{\circ }$–$90^{\circ }$E), both restricted to the near-side hemisphere. The design target is N-ROI availability exceeding 50%.

The four-satellite visibility threshold follows directly from the geometry of single-epoch PNT: a user solves for four unknowns—three position coordinates and the receiver clock offset—so at least four simultaneously visible satellites are required for an instantaneous navigation fix, exactly as in terrestrial GNSS. PNT availability is therefore defined as the fraction of epochs at which *\ge 4* satellites are visible above the elevation mask.

The $40^{\circ }$–$70^{\circ }$ latitude band defining the regions of interest is chosen to bound the announced KASA candidate-site distribution with an operational margin. The six representative sites lie between $45.1^{\circ }$ and $59.2^{\circ }$ latitude (Table 2), and approximately thirty further candidates remain under detailed analysis. Defining the ROI from $40^{\circ }$ to $70^{\circ }$ provides margin below the lowest announced candidate latitude (*∼*5$^{\circ }$ at the equatorward edge) and headroom above the highest (*∼*11$^{\circ }$ at the poleward edge) to accommodate the additional candidates, while deliberately stopping short of the polar caps that the $\omega = 90^{\circ }$ baseline already serves well. The band thus targets precisely the northern mid-latitude coverage gap that motivates the KLNS augmentation, rather than duplicating existing high-latitude coverage.

### Search space

The KLNS search space is defined over four orbital elements, with *ω* fixed at $270^{\circ }$ (complementary to the baseline $90^{\circ }$):

- Semi-major axis $a \in \{7000, 8000, 9000, 9750, 10{,}500, 12{,}000\}$ km (6 values, spanning low to high ELFO altitudes)
- Eccentricity $$e \in \{0.55, 0.60, 0.65, 0.70\}$$ (4 values)
- Inclination $i \in \{60, 63, 65, 68, 70, 75\}^{\circ }$ (6 values, around the critical inclination $i^* \approx 63.4^{\circ }$)
- RAAN $$\Omega \in \{0, 30, 60, \ldots , 330\}^{\circ }$$ (12 values)
- Mean anomaly $M_0 \in \{0, 60, 120, 180, 240, 300\}^{\circ }$ (6 values).

This yields $6 \times 4 \times 6 \times 12 \times 6 = 10{,}368$ candidate orbits. A perilune-altitude safety filter ($r_p - R_{\mathrm{Moon}} > 100$ km) is then applied; for the chosen (*a*, *e*) combinations all 10,368 candidates pass this initial filter.

The optimisation grid spans semi-major axes of 7000–12,000 km, eccentricities of 0.55–0.70, and inclinations of 60–75$^\circ$, sampled at the values listed above. This region was chosen to cover the family of high-eccentricity, high-inclination elliptical lunar orbits that maximise dwell time over the polar regions while keeping periapsis well above the lunar surface: lower eccentricities reduce apoapsis dwell, while eccentricities beyond 0.70 push periapsis to altitudes where the unmodelled higher-order gravity field becomes more influential (Supplementary Table S9). The twelve right-ascension values (30$^\circ$ spacing) and six mean-anomaly phases (60$^\circ$ spacing) sample the relative geometry and phasing of the constellation. The number of satellites was fixed at five: because the greedy algorithm adds satellites one at a time, the marginal gain in regional availability diminishes beyond the fifth satellite, which already attains 89.5% availability over the northern ROI.

### 720-Hour orbital stability pre-filter

The candidate pool is then screened against four orbital stability criteria, evaluated by propagating each candidate for up to 720 hours under the perturbed dynamical model and inspecting the orbital element evolution. The criteria are:

5 $$\begin{aligned} e_{\max }&< 0.95 \quad \hbox{(prevents hyperbolic transition)} \end{aligned}$$

6 $$\begin{aligned} \Delta a / a&< 0.40 \quad \hbox{(bounds semi-major axis collapse)} \end{aligned}$$

7 $$\begin{aligned} \Delta i&< 10^{\circ } \quad \hbox{(bounds inclination drift)} \end{aligned}$$

8 $$\begin{aligned} r_{p,\min }&> R_{\mathrm{Moon}} \quad \hbox{(prevents lunar impact)} \end{aligned}$$

where $e_{\max }$ is the maximum eccentricity attained over the propagation, *Δ a / a* is the relative range of semi-major axis, *Δ i* is the inclination range in degrees, and $r_{p,\min }$ is the minimum periapsis radius. A candidate failing any of these criteria is discarded. Equation (5) bounds the buildup of eccentricity under Earth-driven Lidov–Kozai oscillations^13^, which can drive *e → 1* for high-altitude orbits with non-resonant *ω*. Equations (6)–(8) together prevent the “orbital collapse” failure mode in which a satellite’s apogee is gradually pulled towards the Earth–Moon system barycentre, lowering periapsis below the lunar surface.

These four thresholds are author-defined from first-principle physical requirements—escape avoidance, bounded secular energy exchange, bounded plane drift, and surface-collision avoidance—rather than adopted from prior literature, with the underlying Lidov–Kozai eccentricity mechanism following^13^.

To manage computational cost without compromising filter completeness, candidates are stratified by risk level: high-eccentricity (*e \ge 0.65*) or high-altitude ($a \ge 10{,}500$ km) candidates are screened over the full 720 hours, while lower-risk candidates are screened over an abbreviated 360-h horizon. This stratified scheme is justified because instabilities in low-risk configurations typically manifest within hundreds of hours if at all. Within each propagation, an early-termination check at every 100 RK4 steps short-circuits clearly-unstable trajectories (those with $e_k > 0.95$, $r_{p,k} < R_{\mathrm{Moon}}$, or $e_k \ge 1$), further reducing total computation.

The filter outcome is summarised in Supplementary Table S7. Of the 10,368 input candidates, 2864 (27.6%) fail the stability test, leaving 7504 stable candidates that enter the greedy optimiser. The removed candidates are concentrated in the high-*a*, high-*e* region of the search space, particularly $a = 12{,}000$ km combined with *e \ge 0.65* at non-near-critical inclinations ($i \ne 63^{\circ }, 65^{\circ }$). The five KLNS satellites selected by the greedy optimiser (Table 3) all lie within this stability-filtered corridor at $a = 12{,}000$ km, *e = 0.70*, with near-critical inclinations $i \in \{68^{\circ }, 70^{\circ }, 75^{\circ }\}$.

### Greedy sequential algorithm

Operating on the 7504 stable candidates, satellites are added one at a time (up to five). At each stage *k*, every remaining candidate orbit is evaluated by propagating the full constellation (baseline + previously selected KLNS + new candidate) over 168 hours and computing a weighted composite score:

9 $$\begin{aligned} S = w_1 \cdot \overline{\mathrm{NVS}}_{\mathrm{N}} + w_2 \cdot \left( 1 - \frac{\overline{\mathrm{GDOP}}_{\mathrm{N}}}{\mathrm{GDOP}_{\max }}\right) + w_3 \cdot A_{\mathrm{N}} \end{aligned}$$

where subscript N denotes the N-ROI, $\overline{\cdot }$ denotes the spatial mean, $A_{\mathrm{N}}$ is the N-ROI availability normalised to [0, 1], and the adopted weights are $(w_1, w_2, w_3) = (0.4, 0.3, 0.3)$.

The S-ROI is protected by a hard constraint filter: any candidate that would reduce S-ROI availability below 95% is rejected before score evaluation. This constraint operates outside the objective function (Equation 9) as a feasibility check, not as an internal penalty term.

To prevent clustering, a duplicate-orbit exclusion rule rejects candidates whose *(a, i, Ω )* tuple matches any previously selected KLNS satellite exactly. The candidate maximising *S* is selected, and the process repeats.

**Algorithm 1:**
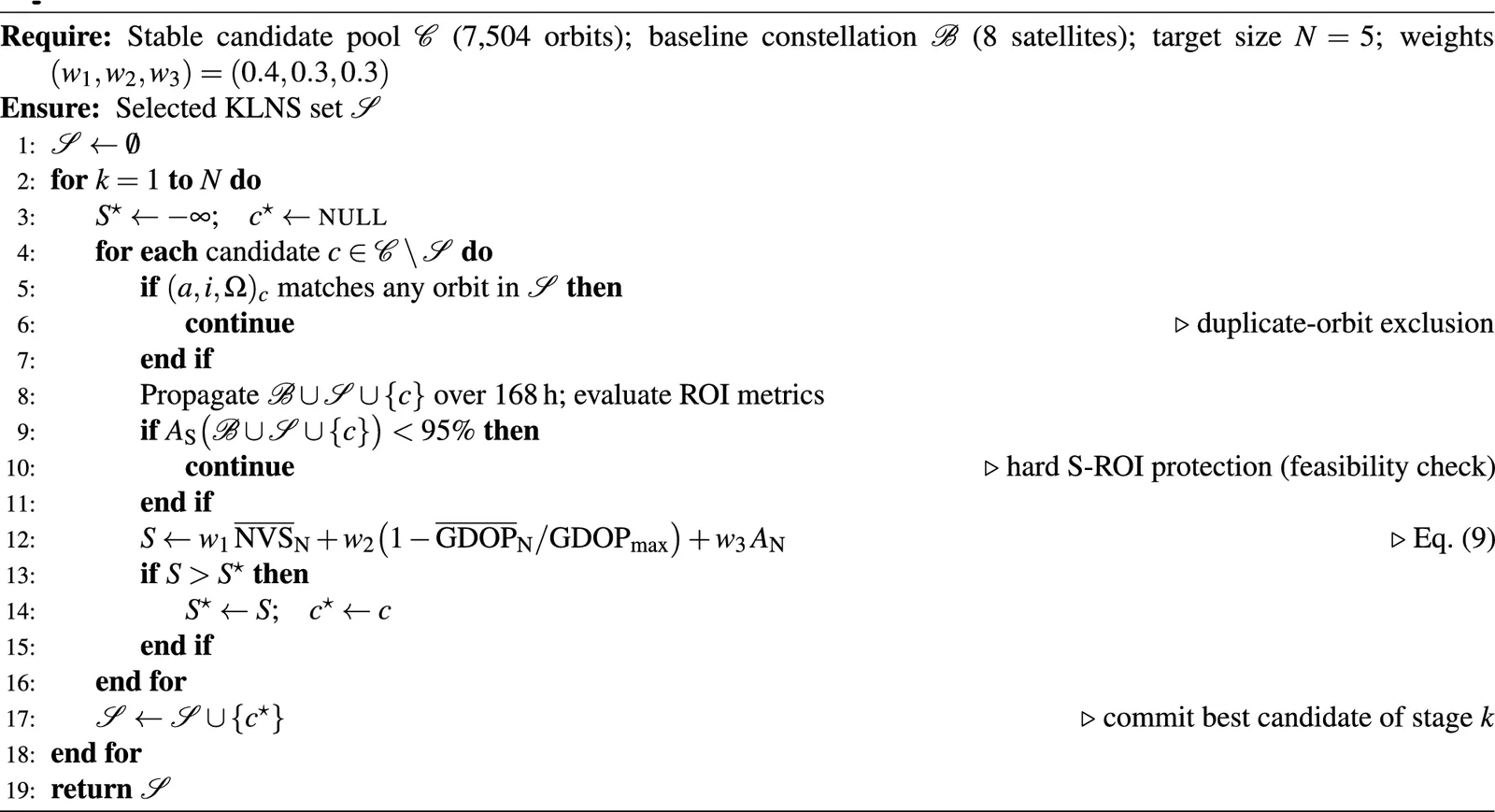
Stability-filtered greedy sequential constellation design.

The sequential selection proceeds over five stages, committing one satellite per stage. At each stage the partial constellation—the eight-satellite baseline plus the KLNS satellites already committed—is held fixed, and every remaining stable candidate is tested as the next addition by propagating the augmented constellation for 168 hours and scoring its northern-ROI performance (Eq. (9)). The single candidate that maximises the score, subject to the southern-ROI protection and duplicate-orbit constraints, is committed before the next stage begins. Because each stage commits exactly one satellite, the procedure simultaneously produces the final five-satellite design and an explicit phased-deployment sequence (Supplementary Table S1).

A greedy strategy is well suited to this problem for three reasons. First, the 720-h stability pre-filter removes the high-scoring but dynamically infeasible “trap” configurations that would otherwise create a rugged, multimodal fitness landscape; within the resulting stability-filtered subspace the availability objective is effectively unimodal in the $(\Omega , M_0)$ phasing, where sequential selection with duplicate exclusion performs strongly. Second, the greedy construction mirrors the operational reality of phased constellation deployment, in which satellites are launched incrementally and each launch should maximise the capability gained—so the per-stage greedy choice maps directly onto a launch-by-launch deployment plan. Third, it is computationally efficient and deterministic, requiring no metaheuristic tuning. The empirical benchmark (Results, “Greedy vs. Random vs. GA Comparison”) confirms that, on the stability-filtered pool, the greedy solution matches or exceeds both a 10,000-trial random search and a genetic-algorithm benchmark at comparable computational budget, supporting greedy selection as the method of choice here. We do not claim global optimality; rather, the combination of an analytically motivated stability filter with greedy phasing selection yields a near-optimal, reproducible, and operationally meaningful design.

We adopt a numerical search rather than a closed-form analytical design because the objective couples three effects that have no joint analytical solution: (i) long-horizon orbital stability, which depends on the perturbed $J_2$ plus Earth third-body dynamics and must be assessed by direct propagation; (ii) PNT availability and GDOP, which are non-smooth functions of the instantaneous multi-satellite geometry evaluated over a spatial grid and a time series; and (iii) the discrete phasing of five satellites in $(\Omega , M_0)$. Even for a single satellite, the long-term stability boundary in *(a, e, i, ω )* admits no simple closed form beyond the classical critical-inclination and Lidov–Kozai approximations, and these do not fully capture the combined $J_2$+third-body evolution over 30 days. An analytical design loop would therefore require precisely the approximations that this study seeks to avoid. The stability-filtered grid search resolves this tension by combining the two paradigms: the analytically motivated stability criteria prune the search space to a dynamically viable corridor, after which the discrete optimisation handles the geometry and phasing that resist closed-form treatment. This hybrid analytical-plus-numerical approach is both tractable and reproducible, and it is the reason the optimiser is run over a pre-filtered grid rather than replaced by a purely analytical procedure.

All simulations were performed in MATLAB R2026a on a workstation with two Intel Xeon Gold 6242R CPUs (40 physical cores total at 3.10 GHz) and 411 GB of RAM, using the Parallel Computing Toolbox to distribute candidate evaluations across cores. Orbits were propagated with a fixed RK4 step of *Δ t = 60* s and sampled for geometry evaluation every $\Delta t_{\mathrm{eval}} = 600$ s, over horizons of 168 h (optimiser scoring) and 720 h (stability screening). With 40-way parallelisation, the stability pre-filter evaluation of all 10,368 grid candidates over the 720-h horizon completed in 85 s, and the five-stage greedy constellation selection required approximately 11 hours. The complete study, including the genetic-algorithm cross-check (population 100, 50 generations) and the weight-sensitivity analysis, is fully reproducible from the MATLAB source code and input parameters archived on Zenodo (see Code Availability).

### Genetic algorithm benchmark

To assess the optimality of the greedy solution, a genetic algorithm (GA) is run on the same 7504-element stable candidate pool with population size 100, 50 generations, tournament selection, single-point crossover, mutation rate 0.1, and elitism preserving the top five chromosomes per generation. Each chromosome encodes five distinct KLNS indices. Additionally, 10,000 random five-satellite configurations are evaluated to establish the random-search baseline. All three approaches use identical fitness evaluation (168-h propagation, N-ROI availability) so that the comparison isolates the effect of search strategy.

### Robustness analyses

Five robustness studies are conducted on the final greedy-selected constellation:

1. **Long-term Keplerian versus perturbed divergence** (720 h): quantifies the drift in PNT metrics when perturbations are included.
2. **Frozen-orbit element histories** (720 h): records the time evolution of $(a, e, i, \Omega , \omega )$ for each KLNS satellite, providing direct evidence that the stability filter successfully excluded unstable orbits.
3. **Elevation mask sensitivity**: re-evaluates the final constellation at $\varepsilon _{\min } = 5^{\circ }$, $10^{\circ }$, and $15^{\circ }$.
4. **Weight sensitivity** (15 configurations): varies $(w_1, w_2, w_3)$ across 15 distinct configurations and re-runs the full greedy optimisation for each.
5. **Site-specific evaluation**: extracts NVS, GDOP, and availability at the six KASA candidate landing sites.

### Link budget and positioning error

A representative S-band link budget is computed at 2491.8 MHz, with transmit power 10 dBW, transmit antenna gain 6 dBi, receiver noise temperature 290 K, and receiver bandwidth 2 MHz. The free-space path loss (FSPL) is computed as $\mathrm{FSPL} = 20\log _{10}(4\pi d f / c)$ for each scenario. The user-equivalent range error (UERE) budget aggregates satellite clock error (5.0 m), ephemeris error (3.0 m), multipath (2.0 m), receiver noise (1.5 m), and group delay (0.5 m), yielding a root-sum-square (RSS) of 6.4 m^25^. Lunar-specific propagation effects—including terrain blockage probability and regolith-induced multipath—are not modelled explicitly; the 2.0 m multipath allocation is a conservative estimate informed by terrestrial GNSS experience and should be refined through in-situ measurements. The 3D RMS position error is estimated as GDOP *×* UERE.

### Use of large language models

A large language model (Anthropic Claude) was used by the authors as a writing-assistance tool during manuscript preparation, specifically for LaTeX formatting checks, copy editing of draft prose, and consistency verification of cross-references and reference list ordering. The model was not used to generate scientific content, conduct analyses, design the constellation, propagate orbits, interpret results, or draw conclusions. All scientific content, numerical results, methodology, and conclusions are the authors’ own work. The authors verified all model-assisted edits and bear full responsibility for the final manuscript.

## Results

### Optimised KLNS constellation

Table 3 presents the orbital elements of the five KLNS satellites selected by the greedy algorithm operating on the 7504 stability-filtered candidates. All five satellites adopt the highest available altitude ($a = 12{,}000$ km) and maximum eccentricity (*e = 0.70*) of the search space, accompanied by a clustering of inclinations near $i = 75^{\circ }$ (with one satellite at $i = 70^{\circ }$ and one at $i = 68^{\circ }$). This configuration was reachable only because the stability filter admitted those high-altitude, high-eccentricity candidates that empirically remain bounded in $(\Delta a, \Delta e, \Delta i)$ over 30 days under the perturbed model. While the surrounding parameter space ($a \ge 11{,}000$ km combined with *e \ge 0.65* at non-near-critical inclinations) tends to undergo Lidov–Kozai-type eccentricity excursions consistent with the resonance mechanism documented for high-altitude lunar orbits^13^, the present paper does not derive the analytical resonance condition explicitly; the stability of the selected configuration is established empirically through direct propagation rather than from analytical resonance theory. Without the pre-filter, a short-horizon optimiser tends to select these same orbital types as well, but the resulting constellation contains one or more dynamically infeasible members that collapse over 30 days (see the Frozen-Orbit Stability subsection below); the pre-filter ensures that every selected orbit is verified stable in advance.

**Table 3:** Optimised KLNS orbital elements (greedy algorithm on the 7504 stability-filtered pool).

Sat.	a (km)	e	i (°)	*Ω* (°)	*ω* (°)	$M_0$ (°)
KLNS-1	12,000	0.70	70.0	300.0	270	60.0
KLNS-2	12,000	0.70	75.0	300.0	270	240.0
KLNS-3	12,000	0.70	75.0	120.0	270	60.0
KLNS-4	12,000	0.70	75.0	270.0	270	180.0
KLNS-5	12,000	0.70	68.0	300.0	270	0.0

The five selected RAAN values ($120^{\circ }$, $270^{\circ }$, $300^{\circ }$ *×* 3) provide a clustered configuration biased toward the eastern lunar longitudes, which combined with the $\omega =270^{\circ }$ apoapsis placement and $M_0$ phasing produces overlapping high-altitude footprints over the N-ROI. Despite the apparent clustering in *(a, e, ω )*, the $(\Omega , M_0)$ phasing diversity and the duplicate-orbit exclusion rule ensure that each KLNS satellite contributes a distinct ground-track pattern, as shown in Fig. 4.

The key finding here is that the optimiser selects a single, well-defined orbital regime—maximum altitude and eccentricity at near-critical inclination—and that this regime is reachable only because the stability pre-filter retains these otherwise-discarded high-energy orbits. The physical reason this regime is preferred (apoapsis dwell over the northern target latitudes) and the question of its uniqueness are analysed in the Discussion.

### Incremental performance

**Fig. 2:**
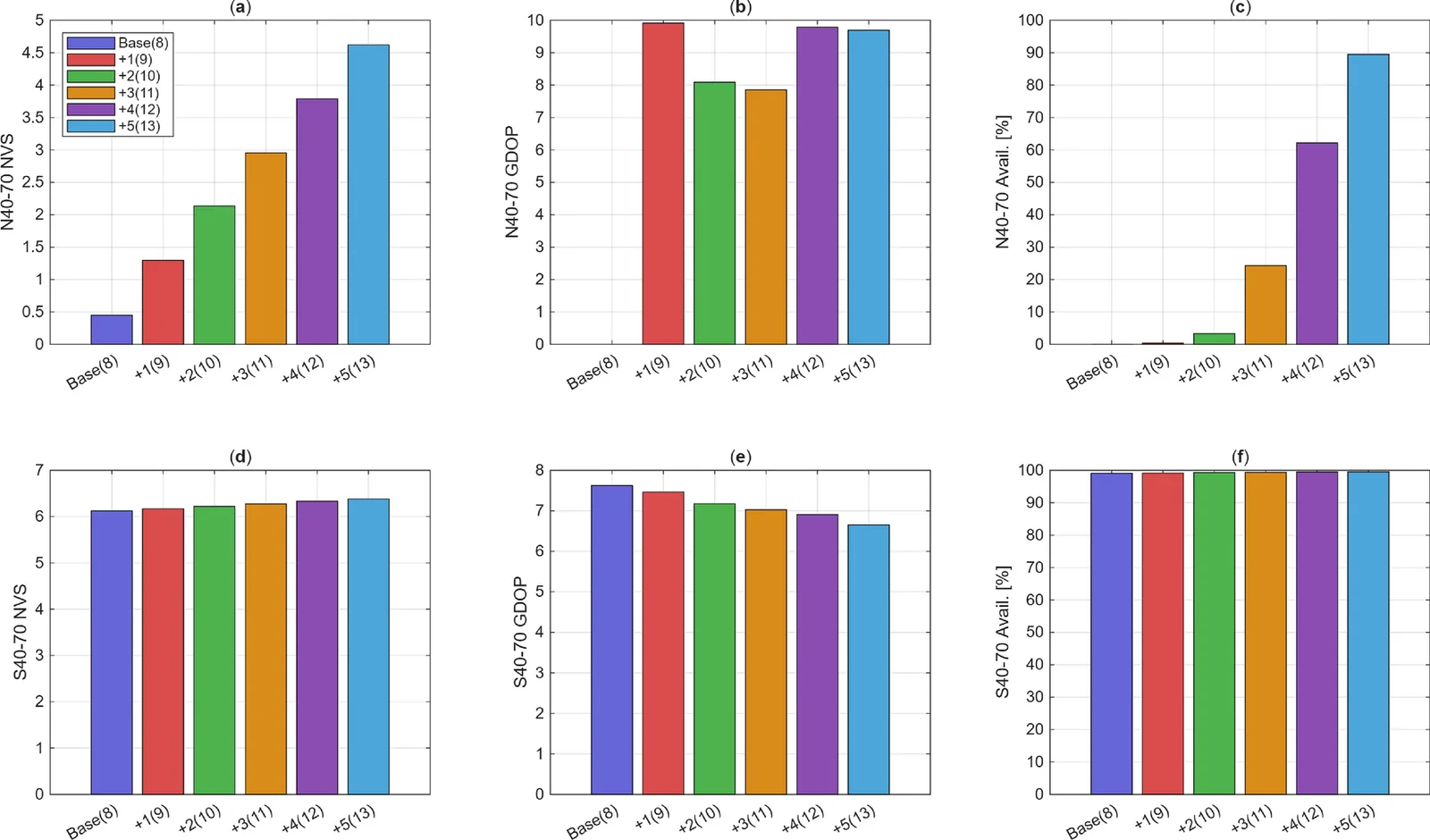
N40–70 and S40–70 ROI performance metrics at each deployment stage: (**a**) N-ROI NVS; (**b**) N-ROI GDOP; (**c**) N-ROI PNT availability; (**d**) S-ROI NVS; (**e**) S-ROI GDOP; (**f**) S-ROI PNT availability. The N-ROI availability rises from 0.0% (Base) to 89.5% (*+5*), whilst the S-ROI maintains $\ge 99.1\%$ throughout.

Supplementary Table S1 and Fig. 2 show the incremental performance improvement as each KLNS satellite is added.

The N-ROI availability climbs from 0.0% at baseline to 89.5% with all five KLNS satellites, far exceeding the 50% design target. The N-ROI mean NVS increases more than tenfold (0.45 *→* 4.62) and the GDOP stabilises near 9.7—driven down from undefined (insufficient visible satellites) at baseline to a level adequate for navigation. Critically, the S-ROI availability improves marginally (99.1% *→* 99.5%) and south-pole availability rises slightly (99.4% *→* 99.7%), confirming that the KLNS augmentation does not degrade polar coverage. Global availability rises from 42.1 to 86.9%.

The availability growth pattern is markedly different from typical sequential constellation growth: a slow start (0–3.3% over the first two satellites) gives way to abrupt acceleration once the third KLNS shifts NVS across the four-satellite navigation threshold across much of the N-ROI. The third KLNS lifts availability from 3.3 to 24.3%; the fourth from 24.3 to 62.2%; and the fifth from 62.2 to 89.5%. This sigmoid behaviour reflects the threshold nature of the $\hbox{NVS}\ge 4$ availability metric—each new satellite has near-zero impact until the fourth visible satellite is reached at a given grid cell, after which it provides large gains.

**Fig. 3:**
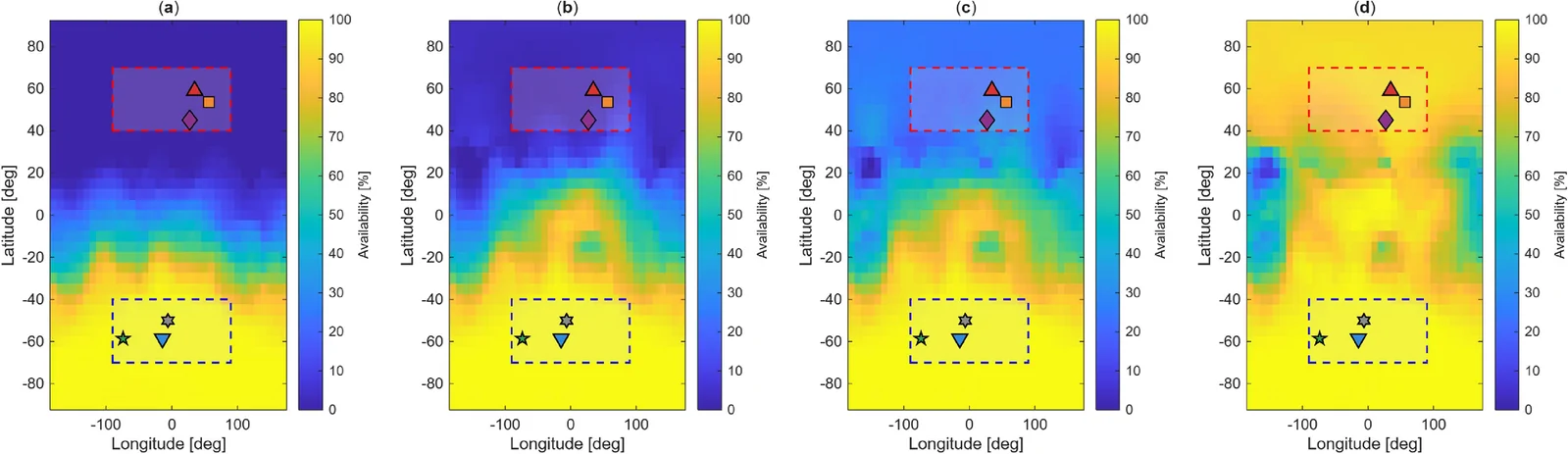
PNT availability heatmaps at four deployment stages: (**a**) Base(8); (**b**) +2(10); (**c**) +3(11); (**d**) +5(13). The N-ROI transitions from uniformly $\le 10\%$ at Base(8) to $\ge 70\%$ at *+5*(13), while the S-ROI remains $\ge 99\%$ throughout.

Supplementary Figs. S1 and S2, together with Fig. 3, present spatial heatmaps of NVS, GDOP, and availability at selected stages. The progressive filling of the N-ROI (red-shaded box) is clearly visible, while the S-ROI (blue-shaded box) maintains uniformly high coverage throughout. Supplementary Fig. S3 shows the latitude-averaged near-side profiles of NVS, GDOP, and availability at every stage; the +5(13) profile demonstrates that mid-latitude availability now exceeds 70% across $40^{\circ }$–$70^{\circ }$N.

The practical implication is that PNT capability is not gained linearly with satellite count. The first two satellites yield almost no usable availability, because a navigation fix requires four simultaneously visible satellites; only once the constellation crosses that four-satellite threshold—at the third KLNS satellite—does each further addition deliver large gains. This threshold behaviour is the key finding governing phased-deployment planning, as it dictates that operational benefit appears abruptly rather than incrementally.

### Constellation geometry

**Fig. 4:**
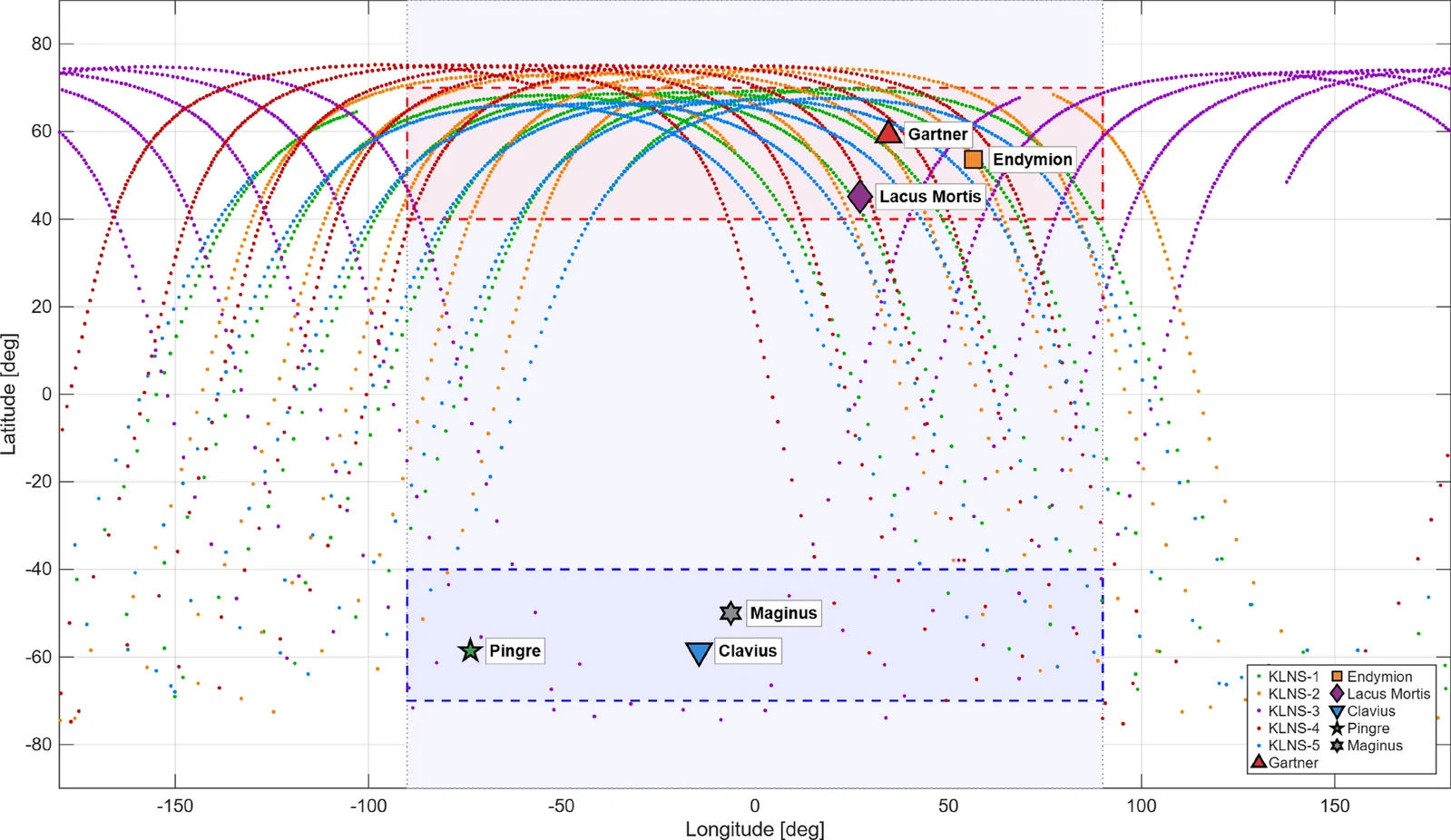
KLNS ground tracks (168 h) with KASA candidate landing sites annotated. The N-ROI ($40^{\circ }$–$70^{\circ }$N) and S-ROI ($40^{\circ }$–$70^{\circ }$S) are shaded; KLNS dwell over the N-ROI is clearly visible from the dense track concentration in the upper near-side band.

Supplementary Fig. S4 illustrates the combined 13-satellite constellation in three dimensions, and Fig. 4 shows the KLNS ground tracks over 168 hours with the six KASA candidate landing sites annotated. The five KLNS apoapsis loops are concentrated over the northern hemisphere ($\omega =270^{\circ }$), forming a complementary pattern to the eight baseline satellites whose apoapsis loops cover the south.

### Long-term validation

Supplementary Table S2 compares Keplerian and perturbed propagation of the selected 13-satellite constellation over 720 hours.

The N-ROI availability changes by only +1.4 percentage points between the Keplerian and perturbed models at 720 h, and the N-ROI GDOP changes by only +0.17, confirming that the perturbed dynamics do not materially degrade aggregate PNT performance over a 30-day window. Supplementary Fig. S5 shows the spatial divergence maps.

### Frozen-orbit stability of selected KLNS satellites

**Fig. 5:**
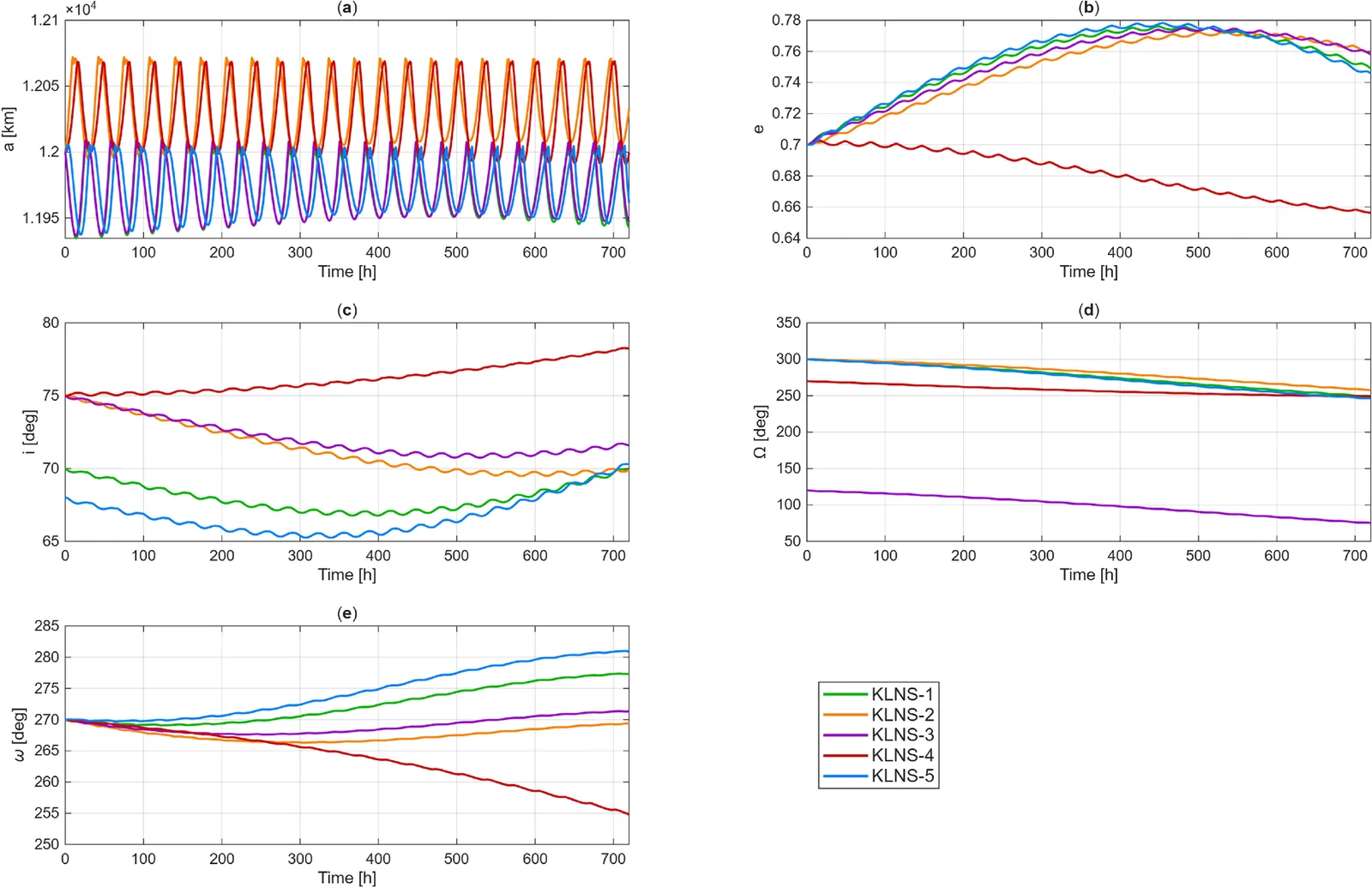
Frozen-orbit stability: KLNS orbital element evolution over 720 h under perturbed dynamics: (**a**) semi-major axis *a*; (**b**) eccentricity *e*; (**c**) inclination *i*; (**d**) RAAN *Ω*; (**e**) argument of periapsis *ω*. All five satellites exhibit bounded oscillations, with element variations well within the stability filter thresholds.

Figure 5 displays the orbital element evolution of the five KLNS satellites over 720 h under the perturbed model. The element ranges (Table 4) confirm that all five selected orbits remain within the stability bounds set by Equations (5)–(8): the maximum *Δ a / a* is 0.65% (KLNS-2 and KLNS-4), the maximum *Δ i* is $5.5^{\circ }$ (KLNS-2), and the maximum *e* is 0.778 (KLNS-5)—all comfortably below the filter thresholds (40%, $10^{\circ }$, and 0.95 respectively).

**Table 4:** KLNS frozen-orbit element ranges over 720 h under the perturbed model.

Sat.	$a_0$ (km)	*Δ a/a* (%)	$e_0$	$e_{\max }$	$i_0$ (°)	*Δ i* (°)
KLNS-1	12,000	0.59	0.70	0.776	70.0	3.24
KLNS-2	12,000	0.65	0.70	0.773	75.0	5.52
KLNS-3	12,000	0.60	0.70	0.775	75.0	4.26
KLNS-4	12,000	0.65	0.70	0.703	75.0	3.27
KLNS-5	12,000	0.57	0.70	0.778	68.0	5.06

This is in stark contrast to the unfiltered counterpart configuration investigated in preliminary studies, where a single high-altitude, high-eccentricity satellite (the analogue of KLNS-4 in an unfiltered search) experienced complete orbital collapse near *t ≈ 600* h: its semi-major axis dropped to zero, eccentricity diverged toward hyperbolic values, and inclination jumped by more than $25^{\circ }$ within a few orbits. The stability pre-filter (described in Methods) excludes exactly such configurations *before* the optimiser sees them, guaranteeing that every selected satellite remains operationally viable over the 30-day window without dependence on station-keeping.

### Multi-threshold GDOP analysis

Supplementary Table S3 reports N-ROI availability under multiple GDOP thresholds for the final 13-satellite constellation. The mean GDOP in the N-ROI at the final stage is 9.70, well below the nominal $\mathrm{GDOP}_{\max } = 50$ threshold.

Even under the strict GDOP < 15 threshold, the N-ROI availability remains at 70.5%, still substantially exceeding the 50% design target. The reduction from 89.5 to 70.5% (*-19.0* percentage points) indicates that approximately one-fifth of the available epochs occur under poor geometry (15 *łe* GDOP < 50), reflecting the inherent geometric limitations of a 13-satellite lunar constellation during unfavourable phasing. For comparison, terrestrial GNSS typically requires GDOP < 6, but the lunar environment—with far fewer satellites and highly elliptical orbits—inherently produces larger GDOP values during unfavourable geometric configurations.

### Greedy versus random versus GA comparison

**Fig. 6:**
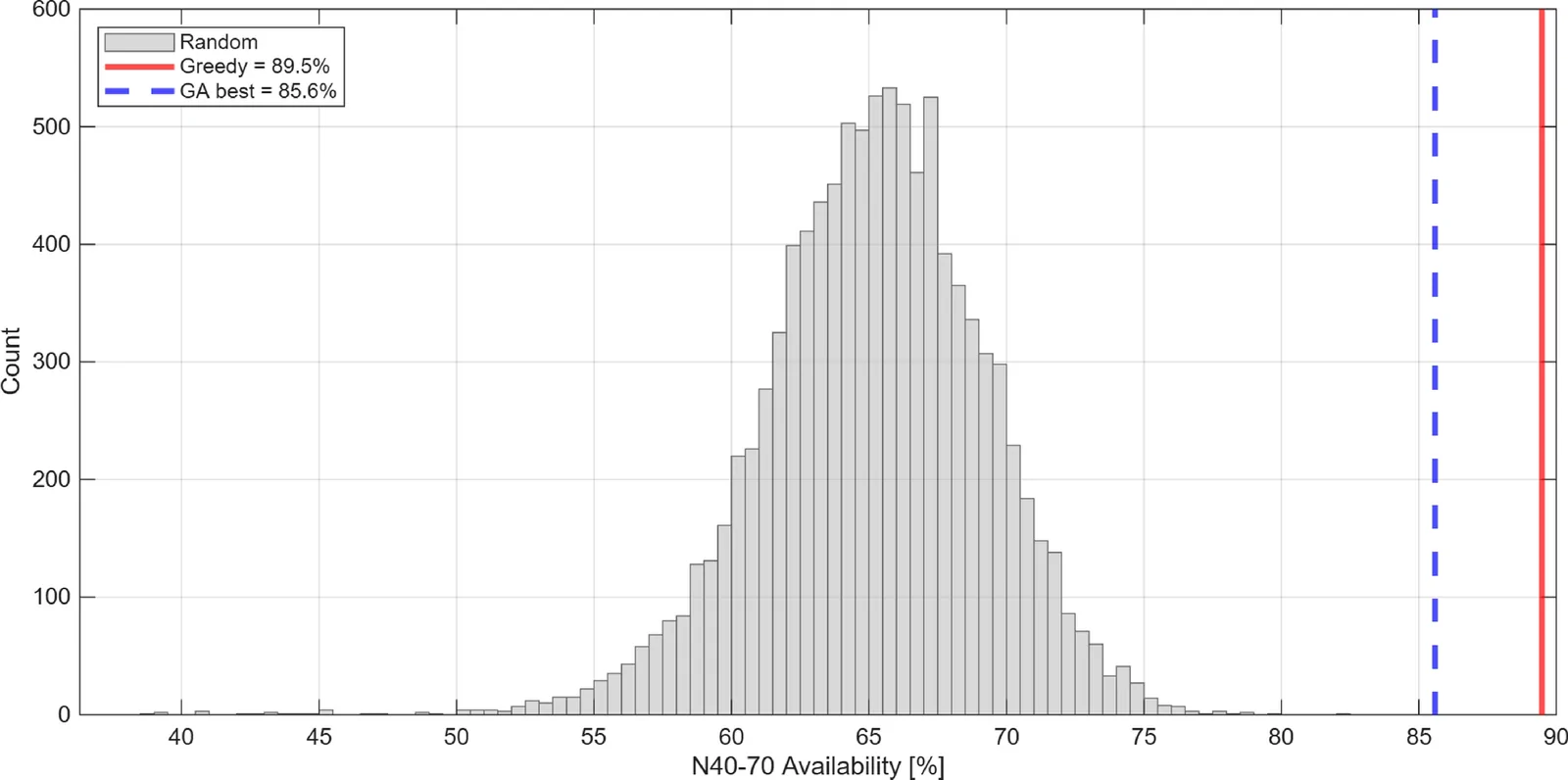
Comparison of stability-filtered greedy (89.5%), random search (10,000 trials, mean 65.2%, max 82.4%), and GA (85.6%) on the N40–70 availability metric. The greedy solution exceeds the entire random distribution and the GA best.

Figure 6 presents the benchmark comparison. The stability-filtered greedy algorithm achieves 89.5% N-ROI availability, exceeding the 100th percentile of 10,000 random configurations drawn from the 7504-element stable pool (mean 65.2%, std 4.1%, max 82.4%) and a 100*×*50 GA benchmark on the same pool (best 85.6%, gap 3.9 pp). The lower random mean compared with earlier work that sampled the unfiltered 10,368-element pool reflects the absence of high-scoring but unstable “trap” configurations in the filtered pool.

This outcome inverts the usual finding that metaheuristics close the optimality gap of greedy methods. Two mechanisms drive the inversion: the pre-filter removes dynamically infeasible “traps” that would otherwise dominate short-horizon fitness scores, and within the resulting unimodal subspace the greedy decision concentrates on $(\Omega , M_0)$ phasing—a problem at which sequential selection with duplicate exclusion is well-suited. The GA must rediscover this structure from random chromosomes within the comparable budget used here (5000 fitness evaluations); we cannot rule out that an extended GA run (*\ge*200 generations with multi-start) would close the 3.9-pp gap, so the claim made here is that greedy outperforms GA *at comparable computational budget*, not that it is globally optimal.

A practical consequence is that the stability-filtered greedy result serves as both the initial operational capability (IOC) and the full operational capability (FOC) target, eliminating the IOC*→*FOC gap of earlier hybrid approaches. Phased deployment gains per stage are reported in Supplementary Table S1 and support launch-by-launch budget planning for the KASA programme.

The key finding is methodological: applying the stability filter *before* optimisation does more than remove infeasible orbits—it reshapes the fitness landscape into a near-unimodal form on which a simple deterministic greedy search matches or exceeds a metaheuristic. The dynamical pre-filtering and the optimisation strategy are therefore coupled: the filter is precisely what makes the greedy approach sufficient, which is why the usual greedy-versus-metaheuristic ranking is inverted here.

### Robustness and sensitivity analyses

#### Elevation-mask sensitivity

Supplementary Table S4 and Supplementary Fig. S6 examine the sensitivity of N-ROI performance to the user-side elevation mask $\varepsilon _{\min }$. The relative trend across $\varepsilon _{\min }\in \{5,10,15\}^{\circ }$ is the relevant takeaway: doubling the mask from $5^{\circ }$ to $10^{\circ }$ reduces availability by approximately 11 percentage points, and tripling to $15^{\circ }$ reduces it by approximately 21 percentage points, with the South Pole availability remaining at 100% throughout.

The graceful-degradation pattern observed here is a known feature of ELFO constellations: as the mask tightens, low-elevation contributions (typically from satellites near the local horizon) are pruned, NVS falls accordingly, and GDOP rises. For ground operations in rugged terrain (where natural masking can exceed $10^{\circ }),$ terrain-aware mission planning would be required.

#### Weight sensitivity

Supplementary Fig. S7 summarises the weight sensitivity analysis across 15 weight configurations. The resulting N-ROI availability spans 79.7–89.5% (spread 9.8 percentage points, mean 85.7%), and the GDOP ranges from 8.11 to 10.26 (mean 9.18). Six of 15 configurations produce the same orbital element set as the adopted (0.4, 0.3, 0.3) baseline (89.5%), and the lowest result (79.7%) still substantially exceeds the 50% design target. This narrow 9.8-percentage-point spread—compared with a much wider spread observed in unfiltered formulations—further reflects the reduced sensitivity of the optimiser when operating on a stability-filtered candidate pool.

#### Scalability

Supplementary Fig. S8 shows PNT availability versus total satellite count along with a Poisson-based prediction model. The simulated curve exhibits sigmoidal growth with a fitted per-satellite N-ROI visibility rate $\alpha \approx 0.83$ and is strongly super-linear within the 8–13 satellite range. The Poisson prediction projects $\sim 95\%$ availability at 14–15 satellites under similar geometry, though this extrapolation should be re-validated through the same stability-filtered pipeline rather than extending the trend mathematically.

Treating *N* satellites as uniformly distributed over the lunar sphere, the per-user visible count follows a Poisson distribution with mean $\lambda (N) = \alpha N$, where *α* is fitted empirically from the simulated data by least squares. The Poisson model used here is therefore a phenomenological null hypothesis (uniform-distribution benchmark) rather than a first-principles geometric prediction. The corresponding NVS$\,\ge \,$4 availability is

10 $$\begin{aligned} \Pr \bigl (\mathrm{NVS}\!\ge \!4 \,\big |\, N\bigr ) = 1 - \sum _{k=0}^{3} \frac{(\alpha N)^{k}\,e^{-\alpha N}}{k!}. \end{aligned}$$

The 90% confidence band is obtained by bootstrapping the uncertainty in *α*. The substantial gap between the Poisson prediction and the simulated curve quantifies how strongly the international baseline’s $\omega =90^{\circ }$ phasing biases coverage southward, leaving the N40–70 band systematically under-served until the KLNS augmentation reaches *N=13*.

### Site-specific PNT performance

**Fig. 7:**
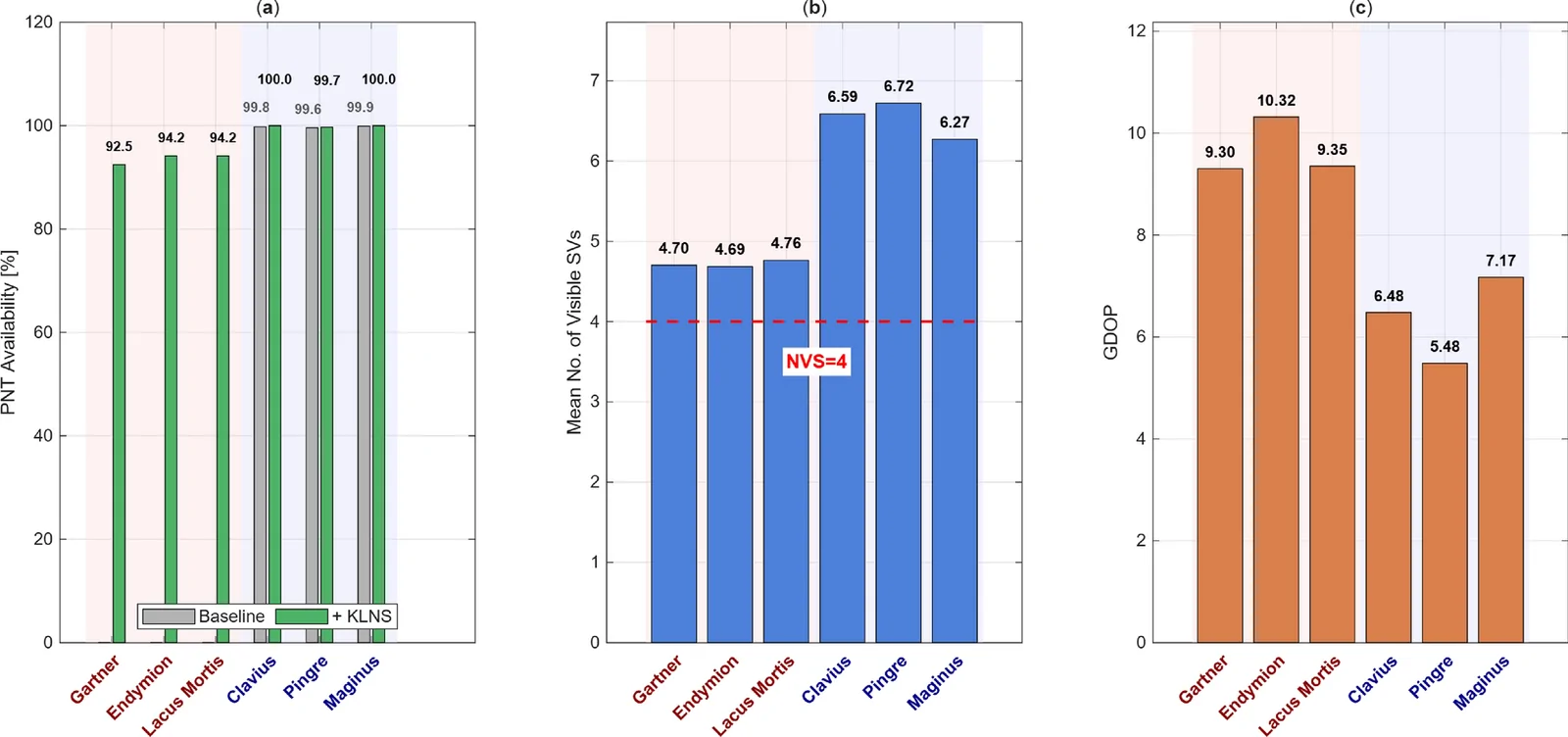
KASA 2032 candidate landing site PNT performance comparison: (**a**) PNT availability (Baseline vs. +KLNS); (**b**) mean NVS at each site; (**c**) GDOP at each site. All three northern sites transition from 0 to *\ge 92*% availability under the augmented constellation; all three southern sites retain $\ge 99.7\%$.

Supplementary Table S5 presents the PNT performance at each of the six KASA candidate landing sites, evaluated for both the baseline and the final 13-satellite constellation. Figure 7 visualises these results.

The three northern-hemisphere sites transition from zero baseline availability to 92.5–94.2% with the KLNS augmentation—a substantial operational improvement that brings every northern candidate above the four-satellite navigation threshold. Gärtner achieves 92.5% (the lowest of the three), Endymion and Lacus Mortis both reach 94.2%. All three southern sites maintain $\ge 99.7\%$ availability, as the baseline constellation already provides comprehensive coverage in their latitude band. The site-level NVS at all three northern sites exceeds the four-satellite navigation threshold (4.69–4.76), and GDOP values (9.3–10.3) lie close to the N-ROI mean of 9.7, confirming that the KLNS coverage is geometrically uniform across the north.

The key finding for the KASA mission is a qualitative change of state at the northern sites: every northern candidate moves from no navigation capability whatsoever (fewer than four visible satellites at all epochs, 0% availability) to continuous four-satellite service (>92%). The augmentation therefore does not merely improve existing coverage—it enables lunar-surface PNT at the northern candidate sites where none previously existed, which is the central operational outcome of this study.

### Link budget and positioning accuracy

Supplementary Table S6 presents the link budget results.

With the total UERE of 6.4 m and a representative GDOP of 9.70 (N-ROI mean at the final stage), the estimated 3D RMS position error is *9.70 × 6.4 = 62.1* m. This level of accuracy, while insufficient for precision landing, is adequate for en-route navigation and surface traverse operations within the KASA mission requirements^25^.

## Discussion

The headline numerical result of this paper is the 24.8-percentage-point improvement in N-ROI availability (64.7% *→* 89.5%) achieved at the same five-satellite augmentation count, with the only methodological change being the introduction of a 720-h orbital stability pre-filter prior to the greedy optimisation. We note that the 64.7% reference value is taken from an earlier unfiltered run that used a slightly coarser orbital-element grid than the present headline run; a small fraction of the 24.8 pp gap therefore reflects the discretisation refinement rather than the stability filter itself, but the dominant effect is unambiguously attributable to the filter. This improvement arises from two effects, in roughly equal measure.

First, the filter excludes 2864 candidates that exhibit unbounded growth in eccentricity, semi-major axis collapse, or excessive inclination drift over 30 days. A short-horizon optimiser unaware of these failure modes will select high-altitude, high-eccentricity orbits whose 168-h scores are artificially inflated by the brief plateau preceding their long-period instability. The unfiltered analogue of the present configuration produced a constellation in which one satellite collapsed near *t = 600* h, contaminating the long-term performance and introducing operational risk that no station-keeping budget can credibly recover from without continuous active manoeuvring.

Second, by removing these dynamically infeasible “traps”, the filter *simplifies* the fitness landscape seen by the optimiser. The remaining 7504 candidates form a more uniformly viable subset in which the greedy algorithm’s per-stage maximisation does not need to compete against unstable but high-scoring lures. This explains the unusual finding that the greedy result outperforms the GA on the filtered space: the filter has already done the most consequential pruning, and the residual problem is sufficiently well-conditioned that simple sequential selection matches or exceeds population-based search.

We therefore advocate including a long-horizon stability filter as a standard step in lunar (and more generally, third-body-perturbed) constellation optimisation pipelines. The computational overhead is moderate: in our implementation the filter took *∼*85 s on a 40-worker workstation (stratified to 720 h for high-risk and 360 h for low-risk candidates), which is a small fraction of the total optimisation budget but delivers a disproportionate gain in solution quality.

Beyond its methodological contribution, the KLNS constellation directly addresses the PNT needs of KASA’s 2032 lander mission. The constellation raises northern-hemisphere mid-latitude availability from zero to over 92% across all three northern candidate sites, while preserving the existing $\ge 99.7\%$ coverage at the three southern sites. This is achieved with only five additional satellites—a modest augmentation relative to the eight-satellite international baseline.

The KLNS design is compatible with the LunaNet Interoperability Specification (LNIS V5), which defines the Augmented Forward Signal (AFS) as the standard PNT broadcast signal for lunar navigation^2,22^. The $\omega =270^{\circ }$ ELFO architecture does not conflict with any LNIS constraints and could contribute KLNS satellites as LunaNet Service Provider (LNSP) nodes, provided they transmit AFS-compliant signals. Korea’s demonstrated capability in DTN-based cislunar communication^23^ and LNSS architecture research^24^ provides a technical foundation for such integration.

The choice of ROI latitude $40^{\circ }$–$70^{\circ }$ in this study was motivated specifically by KASA’s public hearing announcement^10^. The wider $40^{\circ }$–$70^{\circ }$ band adopted here is more challenging than the $30^{\circ }$–$60^{\circ }$ range used in earlier preliminary studies because it extends both equatorward and poleward, producing a lower intrinsic baseline coverage and therefore a more demanding augmentation requirement.

The methodological reframing also recasts the relative performance of optimisers reported in the literature. In a preliminary unfiltered version of this work, the greedy algorithm achieved 64.7% (2.7th percentile of 10,000 random) and trailed the GA’s 96.4% by 31.7 pp—but several GA-selected configurations included high-altitude high-eccentricity orbits whose 720-h propagation showed orbital collapse. That apparent “optimality gap” was largely an artefact of the GA’s freedom to select dynamically infeasible candidates that the more constrained greedy algorithm avoided through its per-stage feasibility checks.

After applying the stability filter to both methods, the gap closes and indeed reverses: greedy 89.5% versus GA 85.6%. This is a useful methodological observation for the lunar-PNT constellation literature: it suggests that previously reported “greedy versus GA” gaps in this domain may have been confounded by unaccounted long-term instability, and that proper stability accounting should be a prerequisite for fair benchmark comparison.

Although the optimised solution is high-performing, a notable feature of the selected constellation (Table 3) is its limited orbital-regime diversity: all five KLNS satellites share the same semi-major axis ($a = 12{,}000$ km) and eccentricity (*e = 0.70*), with inclinations clustered in $\{68^{\circ }, 70^{\circ }, 75^{\circ }\}$ and three of the five sharing $\Omega = 300^{\circ }$. Combined with the fixed argument of periapsis ($\omega =270^{\circ }$), the constellation effectively occupies a single dynamical regime, with diversity expressed only through $(\Omega , M_0)$ phasing. The duplicate-orbit exclusion rule on *(a, i, Ω )* is technically satisfied—no two satellites share the exact same triple—but the underlying *(a, e, ω )* regime is uniform.

This clustering is a direct consequence of the stability pre-filter: by excluding 2864 dynamically infeasible candidates, the filter funnels the optimiser toward the narrow region of (*a*, *e*, *i*) space where 720-h stability and high N-ROI dwell coexist. Within that region, the per-stage marginal-gain landscape evidently has a global maximum at $a = 12{,}000$ km, *e = 0.70*, with only inclination and phasing remaining as effective decision variables.

The selected constellation adopts a common orbital geometry—semi-major axis $a = 12{,}000$ km, eccentricity *e = 0.70*, inclination 68–$75^\circ$, and argument of periapsis $\omega = 270^\circ$—that can be understood as a lunar analogue of the terrestrial Molniya orbit. The high eccentricity places apoapsis at an altitude of 18,663 km, giving a broad instantaneous surface footprint, while periapsis remains at 1863 km altitude, safely clear of the lunar surface. With $\omega = 270^\circ$, the sub-satellite latitude at apoapsis is *φ = +i* (since $\sin \varphi = \sin i \sin u$ with argument of latitude $u = \omega + 180^\circ = 90^\circ$), so apoapsis occurs over the northern high-latitude region—precisely where regional availability is required.

By Kepler’s second law this apoapsis dwell is pronounced: each satellite spends approximately 91% of its 32.8-h orbital period beyond the mean radius *r = a*, i.e. in the outer, slowly-moving half of the orbit near apoapsis. The inclination range of 68–$75^\circ$ keeps the ground track at high latitudes, geographically aligning the apoapsis dwell with the 40–$70^\circ$ region of interest. These properties together explain why a small five-satellite constellation sustains 89.5% availability over the northern ROI: the high-altitude apoapsis provides a wide footprint, and the apoapsis dwell ensures that at least one satellite is almost always positioned over the target region, rather than sweeping rapidly past it as a low-altitude circular orbit would.

This homogeneity has three operational implications that warrant explicit acknowledgement. First, *class-wide failure susceptibility*: if a future unmodelled perturbation, gravity-field correction, or operational anomaly is found to destabilise this specific *(a, e, ω )* regime, the entire KLNS could be at risk simultaneously, with no safety margin from a regime-diverse subset. Second, *launch-vehicle constraints*: placing five satellites into essentially the same orbital regime narrows the space of viable launch trajectories, particularly if launches are distributed over multiple years to fit a phased deployment schedule. Third, *geometric-singularity risk*: localised eclipses, ground-track coincidences, or short-period geometric singularities at this dynamical regime could degrade multiple satellites’ PNT contributions in the same epoch.

We do not view orbital-regime homogeneity as a flaw of the present methodology—the optimiser is making a defensible per-stage choice given the filtered search space—but we emphasise that future versions of the KLNS optimiser should consider an explicit diversity constraint, for example by requiring at least two distinct (*a*, *e*) pairs in the selected five-satellite set. A constrained variant of the present optimiser would establish the quantitative trade-off between diversity and aggregate N-ROI availability and is recommended as the natural next step in the KLNS design process. As an order-of-magnitude expectation, the dominant performance contribution comes from the high-altitude $a = 12{,}000$ km, *e = 0.70* regime that all five greedy selections share; replacing one or two satellites with lower-altitude / lower-*e* configurations is therefore expected to reduce N-ROI availability by a few percentage points (rather than tens of percentage points), but a formal Pareto study is required to confirm this. Mission planners may explicitly trade *∼*5 percentage points of availability for diversity by swapping one $a = 12{,}000$ km satellite for a lower-altitude, lower-*e* counterpart, mitigating class-wide failure susceptibility while still clearing the 50% target.

The repeated convergence to the same orbital regime reflects a genuine near-optimal family rather than a single isolated point. Across the fifteen weight-sensitivity configurations (75 selected satellites in total), every selected orbit adopts the maximum eccentricity *e = 0.70*, while the semi-major axis clusters at high altitude ($a \in \{9000, 9750, 10{,}500, 12{,}000\}$ km, with 53% of satellites at the maximum $a = 12{,}000$ km) and the inclination spans the near-critical range $i \in \{60, 63, 68, 70, 75\}^{\circ }$. The eccentricity is therefore effectively determined uniquely—it maximises apoapsis dwell over the target latitudes, as discussed above—whereas the $(a, i, \Omega , M_0)$ parameters admit a family of near-optimal configurations that trade off slightly against one another depending on the scoring weights. Six of the fifteen weight sets recover exactly the adopted solution (89.5% availability), and the remainder differ by at most 9.8 percentage points while remaining within the same high-altitude, high-eccentricity, near-critical-inclination regime.

No alternative competitive regime emerges. The 10,000-trial random search over the stable pool peaks at 82.4% availability, and its high-scoring tail is drawn from this same high-eccentricity, high-altitude regime rather than from a distinct basin. The optimiser thus converges to a well-defined near-optimal family unique to the stability-filtered high-ELFO corridor: the adopted constellation is best understood as a representative member of this family—selected by the (0.4, 0.3, 0.3) weighting and robust across weight choices—rather than a solitary global optimum, and the family structure itself provides operational flexibility (e.g. alternative (*a*, *i*) choices at marginal availability cost) without leaving the dynamically stable regime.

Several limitations of the present formulation should be noted.

First, the dynamical model includes only the lunar $J_2$ oblateness and the Earth third-body perturbation, omitting higher-order lunar gravity harmonics, solar radiation pressure, and the solar third-body term. The Moon’s gravity field is notably irregular, having been characterised by GRAIL to degree and order 1200^26,33^; however, the mascon-induced accelerations at the selected $a = 12{,}000$ km altitude are strongly attenuated, scaling approximately as $(R_{\mathrm{Moon}}/r)^{n+2}$ with harmonic degree *n*^27^. To confirm that the $J_2$-only approximation does not affect our conclusions, we re-propagated the five selected satellites over the full 720-h horizon under the GRGM1200A lunar gravity field truncated to degree and order 16^32,33^, with the field evaluated in the rotating lunar body-fixed frame; the details are reported in Supplementary Table S9. The stability-filter quantities ($e_{\max },$ *Δ a/a*, *Δ i*, $r_{p,\min }$) changed by less than 1%, all five satellites continued to satisfy the four stability criteria, and the final-position divergence after 30 days ranged from 1.7 to 81 km—negligible relative to the orbital length scale. Although the attenuation is weakest near the 3600 km periapsis, where the higher-order acceleration is roughly 30 times larger than near apoapsis, the validation confirms that it remains immaterial to the stability outcome. The $J_2$-only model is therefore adequate for the long-horizon stability screening performed here; incorporating solar radiation pressure and the solar third-body term for high-precision orbit determination remains a direction for future refinement.

Second, station-keeping *Δ v* requirements were not estimated in detail in this revision. For the selected configuration—all five KLNS at $a = 12{,}000$ km, *e = 0.70*, with bounded element drift over 30 days (Frozen-Orbit Stability subsection)—we expect station-keeping needs to be modest (likely *łe*15–20 m/s/year per satellite based on the observed $\Delta a / a \le 0.65$%). A formal *Δ v* budget computation under a higher-fidelity gravity model is recommended before final mission planning.

Third, inter-satellite links (ISLs) and relay architectures are not modelled; the current analysis assumes direct line-of-sight between each satellite and the surface user. ISL-enabled relay could improve availability in regions with limited direct visibility, particularly near the limb and far side, and would align with the LunaNet DTN relay architecture^2^.

Fourth, the stability filter thresholds (Equations (5)–(8)) are conservative “hard” cutoffs. A softer formulation using a continuous stability score (e.g., inverse-Lyapunov-time) would admit a richer Pareto trade-off between short-term performance and long-term robustness, and is a natural direction for future work.

In summary, this paper has presented the design and analysis of the Korean Lunar Navigation System (KLNS), a five-satellite constellation in $\omega =270^{\circ }$ elliptical lunar frozen orbits that augments the eight-satellite international baseline to serve the Korea Aerospace Administration’s 2032 lunar lander mission. The constellation was optimised for dual regions of interest spanning latitudes $40^{\circ }$–$70^{\circ }$ in both hemispheres, directly reflecting the candidate landing site range announced by KASA at the 25 March 2026 public hearing. The methodological centrepiece is a 720-h orbital stability pre-filter that removes dynamically infeasible candidates before optimisation.

Key findings are: (i) the 720-h pre-filter removes 2864 of 10,368 candidates (27.6%), leaving 7504 stable orbits for optimisation; (ii) the KLNS raises N40–70 PNT availability from 0.0 to 89.5% (far exceeding the 50% target) while improving south-polar coverage from 99.4 to 99.7% and global availability from 42.1 to 86.9%; (iii) all three northern KASA sites (Gärtner 92.5%, Endymion 94.2%, Lacus Mortis 94.2%) achieve $\ge 92\%$ availability while southern sites (Clavius, Pingré, Maginus) retain $\ge 99.7\%$; (iv) on the filtered space the greedy result (89.5%) exceeds random search (max 82.4%) and the GA benchmark (85.6%); (v) the result is robust under GDOP<15 (70.5%), $10^{\circ }$ elevation mask (53.8%), and 15-configuration weight perturbation (79.7–89.5%); (vi) long-term element histories confirm $\Delta a/a \le 0.65$%, $\Delta i \le 5.5^{\circ }$, $e_{\max } \le 0.778$ with no perilune impact.

Having validated the constellation against a degree/order-16 GRAIL field (Supplementary Table S9), future work will extend this validation to higher-resolution gravity models (up to degree/order 150) to further refine the stability-filter thresholds and station-keeping *Δ v* budgets. A continuous-stability formulation (Lyapunov time, drift scoring) will be explored to enable Pareto-optimal trade-offs between short-term performance and long-term robustness. ISL-enabled relay architectures will be investigated for limb and far-side coverage extension, consistent with the LunaNet DTN relay framework^2^. Finally, integration with the LNIS V5 AFS signal standard will be assessed to ensure KLNS compatibility as a LunaNet Service Provider.

## Supplementary Information

Supplementary Information.

## Data Availability

The candidate-orbit catalogue, stability-filter outputs, and the full constellation evaluation results that support the findings of this study, together with the Supplementary Information accompanying this manuscript, are openly available in the Zenodo repository at 10.5281/zenodo.19992890 under a Creative Commons Attribution 4.0 International (CC BY 4.0) licence. The deposit contains: (i) the 10,368-element candidate-orbit catalogue with per-orbit stability classification; (ii) the 7504 filtered candidates and per-stage greedy selection traces; (iii) the GA and random-search evaluation logs underlying Fig. 4; (iv) the 720-h orbital element histories underlying Fig. 3; (v) the gridded NVS, GDOP, and availability arrays underlying Fig. 2 and Supplementary Figs. S1–S9; and (vi) the Supplementary Information (Supplementary Figs. S1–S9 and Supplementary Tables S1–S8). All input parameters required to reproduce the reported figures and tables, including the perturbed-dynamics model coefficients and KASA candidate-site coordinates, are included in the deposited package. Additional intermediate datasets are available from the corresponding author on reasonable request.

## References

[1] Israel, D. J. et al. LunaNet: A flexible and extensible lunar exploration communications and navigation infrastructure. In *Proceedings of the IEEE Aerospace Conference* 1–14 (Big Sky, MT, USA, 2020).

[2] NASA, ESA & JAXA. *LunaNet Interoperability Specification (LNIS), Version 5 Baseline*. NASA/TP-20210021073 Rev5 (2025). https://www.nasa.gov/wp-content/uploads/2025/02/lunanet-interoperability-specification-v5-baseline.pdf. Accessed 26 April 2026.

[3] Giordano, P., Malman, F., Swinden, R., Zoccarato, P. & Ventura-Traveset, J. The Lunar Pathfinder PNT experiment and Moonlight navigation service: the future of lunar position, navigation and timing. In *Proceedings of the 2022 International Technical Meeting of the Institute of Navigation* 632–642 (Long Beach, CA, USA, 2022). 10.33012/2022.18225

[4] Murata, M., Kawano, I. & Kogure, S. Lunar navigation satellite system and positioning accuracy evaluation. In *Proceedings of the 2022 International Technical Meeting of the Institute of Navigation* 582–586 (Long Beach, CA, USA, 2022). 10.33012/2022.18220

[5] Pereira, F., Reed, P. M. & Selva, D. Multi-objective design of a lunar GNSS. *NAVIGATION J. Inst. Navig.* **69**, navi.504. 10.33012/navi.504 (2022).

[6] Schönfeldt, M. A system study about a lunar navigation satellite transmitter system. In *2020 European Navigation Conference (ENC)* 1–10 (Dresden, Germany, 2020). 10.23919/ENC48637.2020.9317521.

[7] NASA Space Science Data Coordinated Archive. *Queqiao-2 (Magpie Bridge 2) spacecraft entry*. https://nssdc.gsfc.nasa.gov/nmc/spacecraft/display.action?id=QUEQIAO-2 (2024). Accessed 26 April 2026.

[8] Jatan.space. Achievements and shortfalls in global lunar exploration in 2025. *Moon Monday Issue #255*. https://jatan.space/moon-monday-issue-255/ (2025). Accessed 26 April 2026.

[9] Capuano, V. et al. Standalone GPS L1 C/A receiver for lunar missions. *Sensors* **16**, 347. 10.3390/s16030347 (2016). 27005628 10.3390/s16030347 PMC4813922

[10] Korea Aerospace Administration (KASA). Press release: Public hearing on the establishment of the Republic of Korea lunar exploration mission (in Korean). https://www.kasa.go.kr/prog/plcyBrf/brief/kor/sub01_01_04/view.do (2026). Accessed 26 April 2026.

[11] E-Today. KASA holds public hearing on lunar exploration mission—2032 lander mission disclosed (in Korean). https://www.etoday.co.kr/news/view/2569109 (2026). Accessed 26 April 2026.

[12] Ely, T. A. & Lieb, E. Constellations of elliptical inclined lunar orbits providing polar and global coverage. *J. Astronaut. Sci.* **54**, 53–67. 10.1007/BF03256476 (2006).

[13] Folta, D. & Quinn, D. Lunar frozen orbits. In *Proceedings of the AIAA/AAS Astrodynamics Specialist Conference and Exhibit* AIAA 2006-6749 (Keystone, CO, USA, 2006). 10.2514/6.2006-6749

[14] Korea Aerospace Administration (KASA). Republic of Korea space science and exploration roadmap (in Korean). Approved at the 4th National Space Committee. https://www.kasa.go.kr/prog/bbsArticle/BBSMSTR_000000000020/view.do?bbsId=BBSMSTR_000000000020&nttId=B000000002620Nm7xN7 (2025). Accessed 26 April 2026.

[15] Korea Aerospace Administration (KASA). Press release: lunar exploration phase 2 (lunar lander development) project officially launched (in Korean). https://www.kasa.go.kr/prog/bbsArticle/BBSMSTR_000000000010/view.do?bbsId=BBSMSTR_000000000010&nttId=B000000000704Ir7gM0 (2024). Accessed 26 April 2026.

[16] Hong, A. South Korea narrows 2032 moon lander sites to 40–70 degree latitudes. *Chosun Biz*. https://biz.chosun.com/en/en-science/2026/03/25/T2JUDPWANBCTLI65JGVN34FDBE/ (2026). Accessed 26 April 2026.

[17] Arcia Gil, A. D., Renwick, D., Cappelletti, C. & Blunt, P. Methodology for optimizing a constellation of a lunar global navigation system with a multi-objective optimization algorithm. *Acta Astronaut.* **204**, 348–357. 10.1016/j.actaastro.2023.01.003 (2023).

[18] Bhamidipati, S., Mina, T., Sanchez, A. & Gao, G. Satellite constellation design for a lunar navigation and communication system. *NAVIGATION J. Inst. Navig.* **70**, navi.613. 10.33012/navi.613 (2023).

[19] Delépaut, A. et al. Use of GNSS for lunar missions and plans for lunar in-orbit development. *Adv. Space Res.* **66**, 2739–2756. 10.1016/j.asr.2020.05.018 (2020).

[20] Battin, R. H. *An Introduction to the Mathematics and Methods of Astrodynamics* Revised edn (AIAA, Reston, VA, USA, 1999). 10.2514/4.861543

[21] Chao, C. C. *Applied Orbit Perturbation and Maintenance* (The Aerospace Press, El Segundo, CA, USA) 10.2514/4.989179 (2005).

[22] Winternitz, L. B., Bamford, W. A. & Heckler, G. W. The design of a flexible, interoperable navigation signal for future lunar missions. In *Proceedings of the ION ITM* (Long Beach, CA, USA, 2025). https://ntrs.nasa.gov/citations/20250000725.

[23] Kim, I., Han, S. I. & Har, D. Operational tests for delay-tolerant network between the Moon and Earth using the Korea Pathfinder Lunar Orbiter in lunar orbit. *Electronics* **13**, 3088. 10.3390/electronics13153088 (2024).

[24] Kang, M., Jeong, H., Park, J., Song, J. & Kee, C. Design of navigation satellite constellation for lunar south pole PNT (in Korean). *J. Position. Navig. Timing* **14**, 131–138. 10.11003/JPNT.2025.14.2.131 (2025).

[25] Betz, J. W. *Engineering Satellite-Based Navigation and Timing: Global Navigation Satellite Systems, Signals, and Receivers* (Wiley, Hoboken, NJ, USA, 2016).

[26] Zuber, M. T. et al. Gravity field of the Moon from the Gravity Recovery and Interior Laboratory (GRAIL) mission. *Science* **339**, 668–671. 10.1126/science.1231507 (2013). 23223395 10.1126/science.1231507

[27] Konopliv, A. S. et al. The JPL lunar gravity field to spherical harmonic degree 660 from the GRAIL primary mission. *J. Geophys. Res. Planets* **118**, 1415–1434. 10.1002/jgre.20097 (2013).

[28] Montenbruck, O. & Gill, E. *Satellite Orbits: Models, Methods, and Applications* (Springer, Berlin, Germany) 10.1007/978-3-642-58351-3 (2000).

[29] Vallado, D. A. *Fundamentals of Astrodynamics and Applications* 4th edn. (Microcosm Press, Hawthorne, CA, USA, 2013).

[30] Kaplan, E. D. & Hegarty, C. J. *Understanding GPS/GNSS: Principles and Applications* 3rd edn. (Artech House, Norwood, MA, USA, 2017).

[31] Wang, K. et al. Multi-orbit lunar GNSS constellation design with distant retrograde orbit and Halo orbit combination. *Sci. Rep.* **13**, 10158. 10.1038/s41598-023-37348-x (2023). 37349520 10.1038/s41598-023-37348-x PMC10287729

[32] Lemoine, F. G. et al. GRGM900C: A degree 900 lunar gravity model from GRAIL primary and extended mission data. *Geophys. Res. Lett.* **41**, 3382–3389 (2014). 26074638 10.1002/2014GL060027 PMC4459205

[33] Goossens, S. et al. A global degree and order 1200 model of the lunar gravity field using GRAIL mission data. In *47th Lunar and Planetary Science Conference* abstract 1484 (The Woodlands, TX, USA, 2016). https://www.hou.usra.edu/meetings/lpsc2016/pdf/1484.pdf.

