# Supplementary material for: Korean lunar navigation constellation with stability-filtered optimisation
Source: Sci Rep. 2026 Jul 8;16:26657. doi: 10.1038/s41598-026-60190-w (PMC13507044; doi:10.1038/s41598-026-60190-w)
Supplement: Supplementary file 1 [file 41598_2026_60190_MOESM1_ESM.pdf]

Supplementary Information for: Korean Lunar Navigation Constellation with Stability-Filtered Optimisation

Seungsoo Yoo[1], Gyu-In Jee[1], and Sun Yong Kim[1,*]

[1]Konkuk University, Department of Electrical and Electronics Engineering, Seoul, 05029, Republic of Korea
[*]kimsy@konkuk.ac.kr

ABSTRACT

This Supplementary Information accompanies the manuscript “Korean Lunar Navigation Constellation with Stability-Filtered Optimisation” and provides additional figures, tables, and validation notes that complement the main text. The Supplementary Figures (S1–S8) include spatial heatmaps of the number of visible satellites and geometric dilution of precision, near-side latitude profiles, the three-dimensional baseline+KLNS constellation geometry, Keplerian-versus-perturbed divergence maps, elevation-mask sensitivity, weight sensitivity, and scalability analysis. The Supplementary Tables (S1–S9) include the per-stage incremental performance, long-term Keplerian-versus-perturbed comparison, multi-threshold GDOP availability, elevation-mask sensitivity, site-specific PNT performance, link budget, the stability-filter outcome counts, the RK4 step-size convergence study, and the degree/order-16 gravity-field validation. The Supplementary Notes provide the RK4 step-size convergence analysis and the higher-order gravity-field validation supporting the main-text dynamical model. Items are numbered in the order they appear in the corresponding section of the main manuscript.

Supplementary Figures

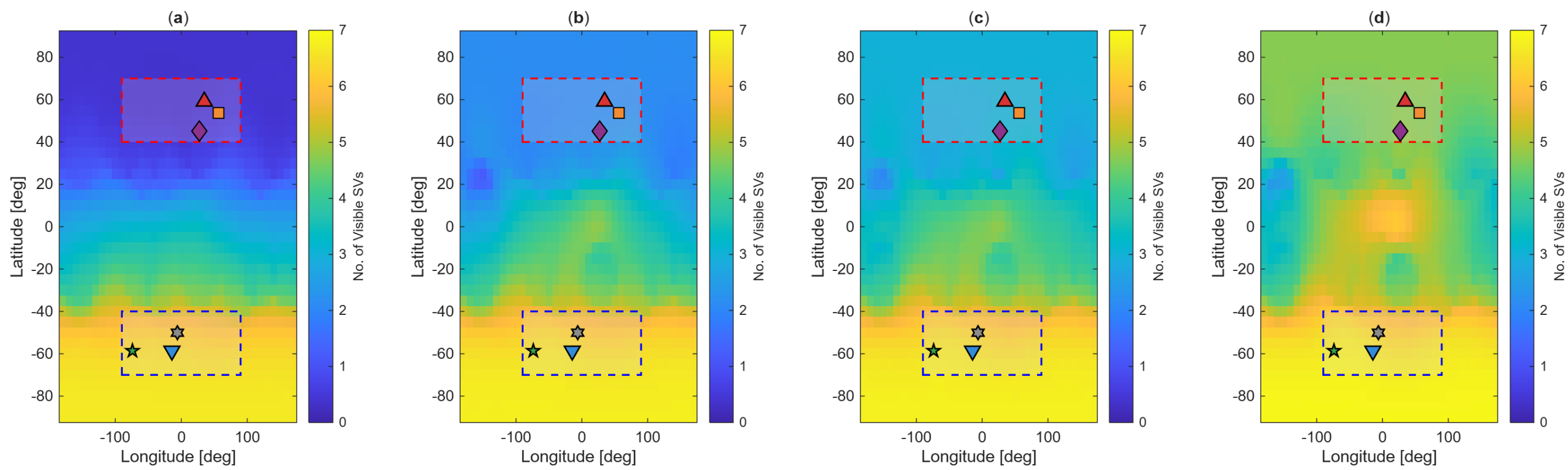

Figure S1. NVS heatmaps at four deployment stages: (**a**) Base(8); (**b**) +2(10); (**c**) +3(11); (**d**) +5(13). KASA candidate landing sites are marked with symbols.

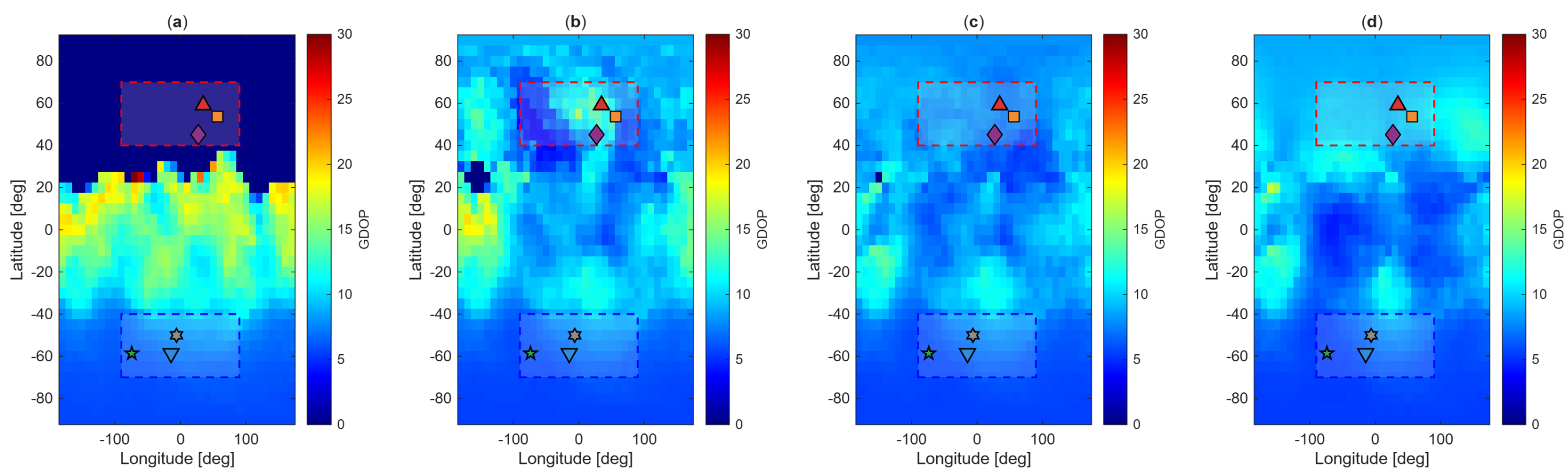

Figure S2. GDOP heatmaps at four deployment stages: (**a**) Base(8); (**b**) +2(10); (**c**) +3(11); (**d**) +5(13). Values below 15 (yellow–green) indicate good positioning geometry; the final stage (+5) shows almost the entire near-side at GDOP≤15 except a residual band near 30°N.

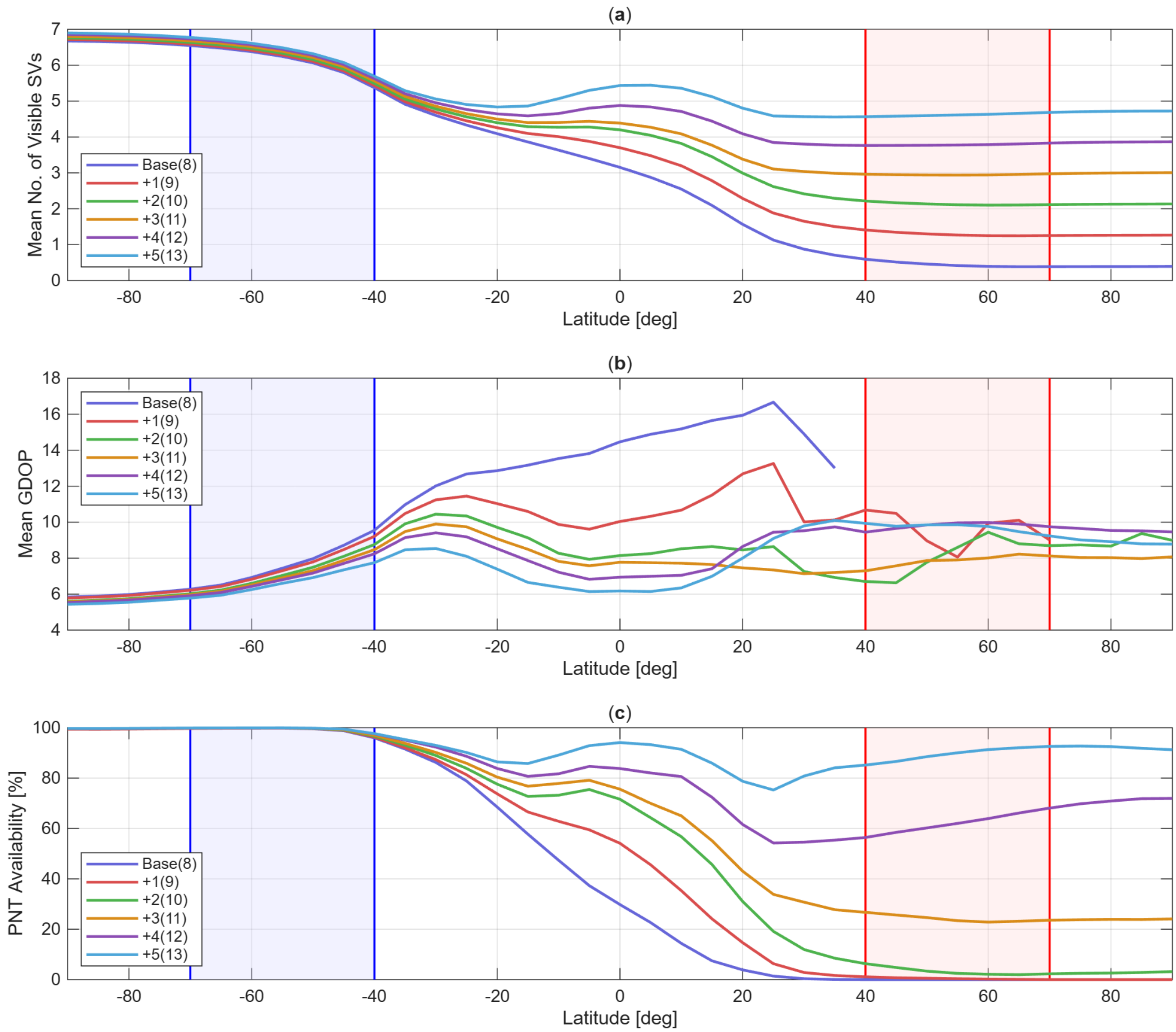

Figure S3. Near-side longitude-averaged latitude profiles at every deployment stage: (**a**) mean NVS; (**b**) mean GDOP; (**c**) PNT availability. The N-ROI (40°–70°N) is shaded in red and the S-ROI (40°–70°S) is shaded in blue. In panel (**b**), curves terminate where the longitude-averaged GDOP becomes undefined because more than half of the longitude cells in that latitude bin have NVS<4 at most epochs: this occurs above ∼28°N for Base(8) and above ∼35°N for +1(9), highlighting precisely the mid-latitude geometry deficit that the KLNS augmentation is designed to close.

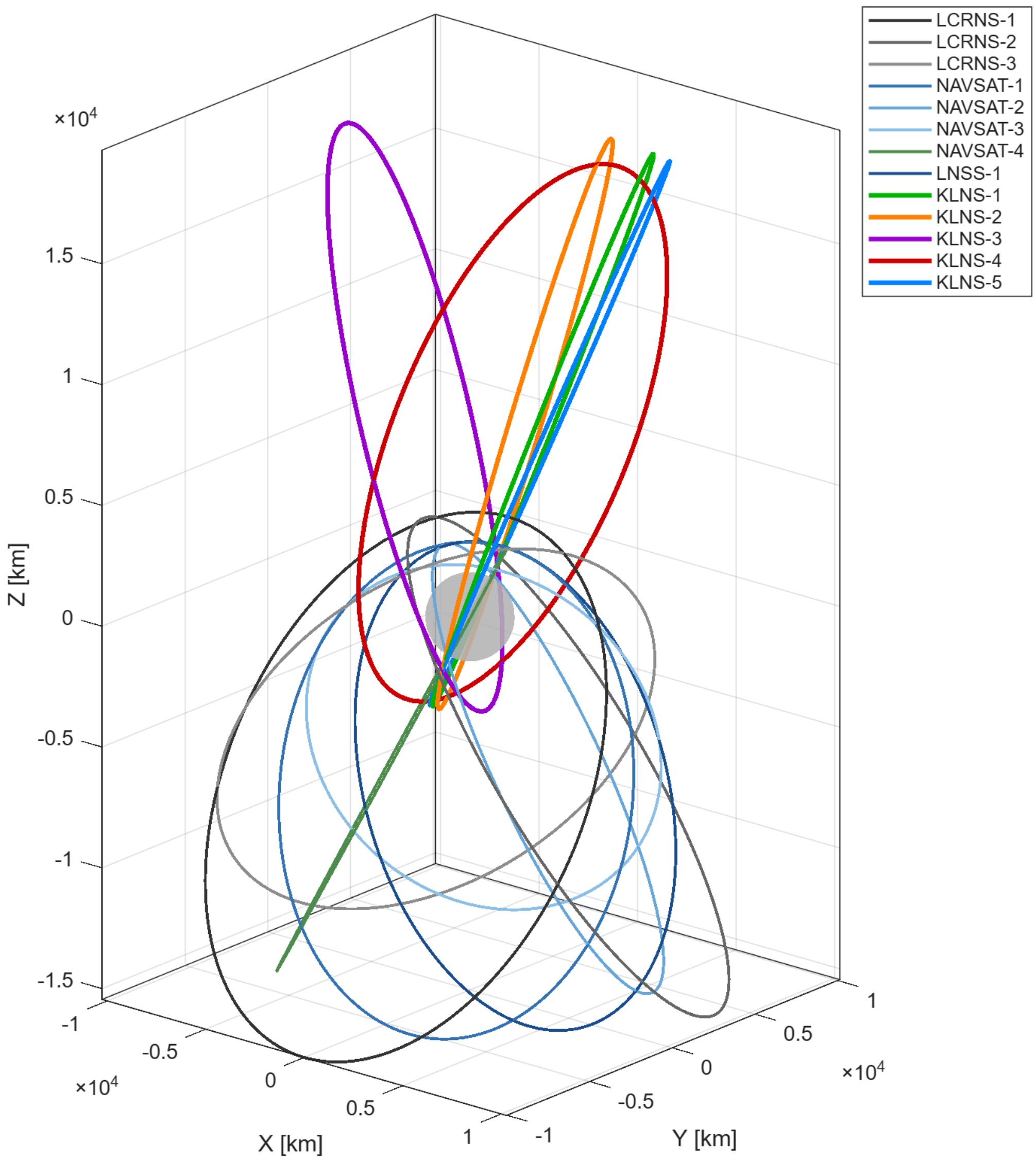

Figure S4. Three-dimensional orbit plot of the baseline (8) + KLNS (5) constellation over one orbital period. The five KLNS orbits (high-altitude ELFOs with $\omega = 270°$) loop over the northern hemisphere, complementing the southern-hemisphere apoapsis of the baseline.

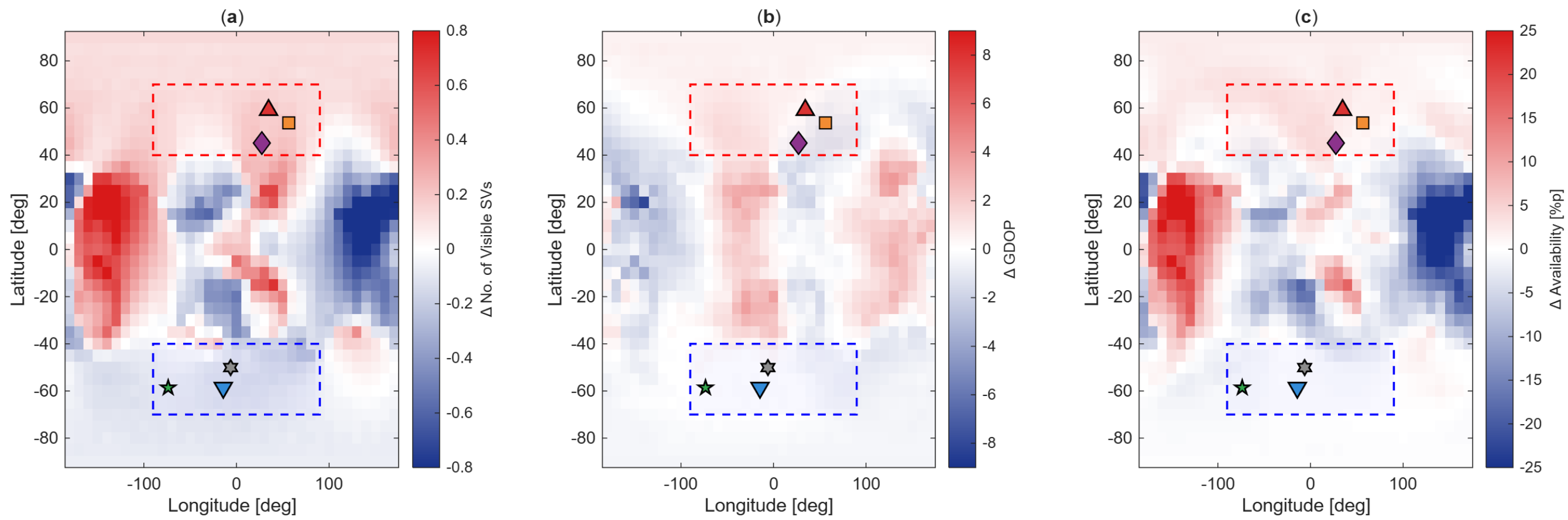

Figure S5. Keplerian vs. perturbed divergence at 720 h: (**a**) ΔNVS; (**b**) ΔGDOP; (**c**) ΔAvail. The differences are spatially patchy but bounded: $|\Delta \text{NVS}| \leq 0.8$, $|\Delta \text{GDOP}| \leq 9$, $|\Delta \text{Avail}| \leq 25$ percentage points at any single grid cell.

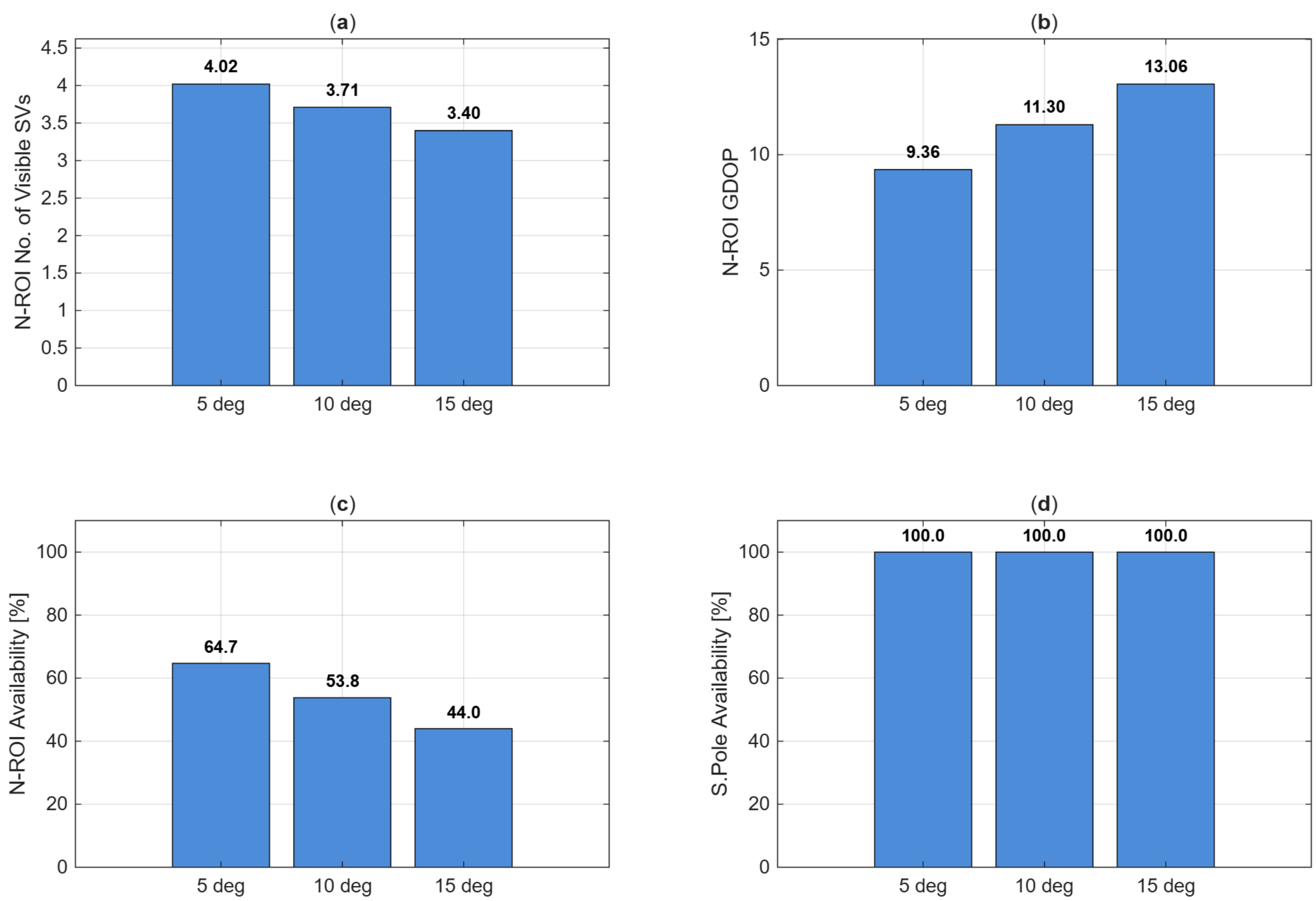

Figure S6. Elevation mask sensitivity for the 13-satellite constellation: (**a**) N-ROI mean NVS; (**b**) N-ROI mean GDOP; (**c**) N-ROI PNT availability; (**d**) South Pole availability. The N-ROI availability degrades gracefully from 64.7% at 5° to 44.0% at 15°; the South Pole availability remains 100% throughout.

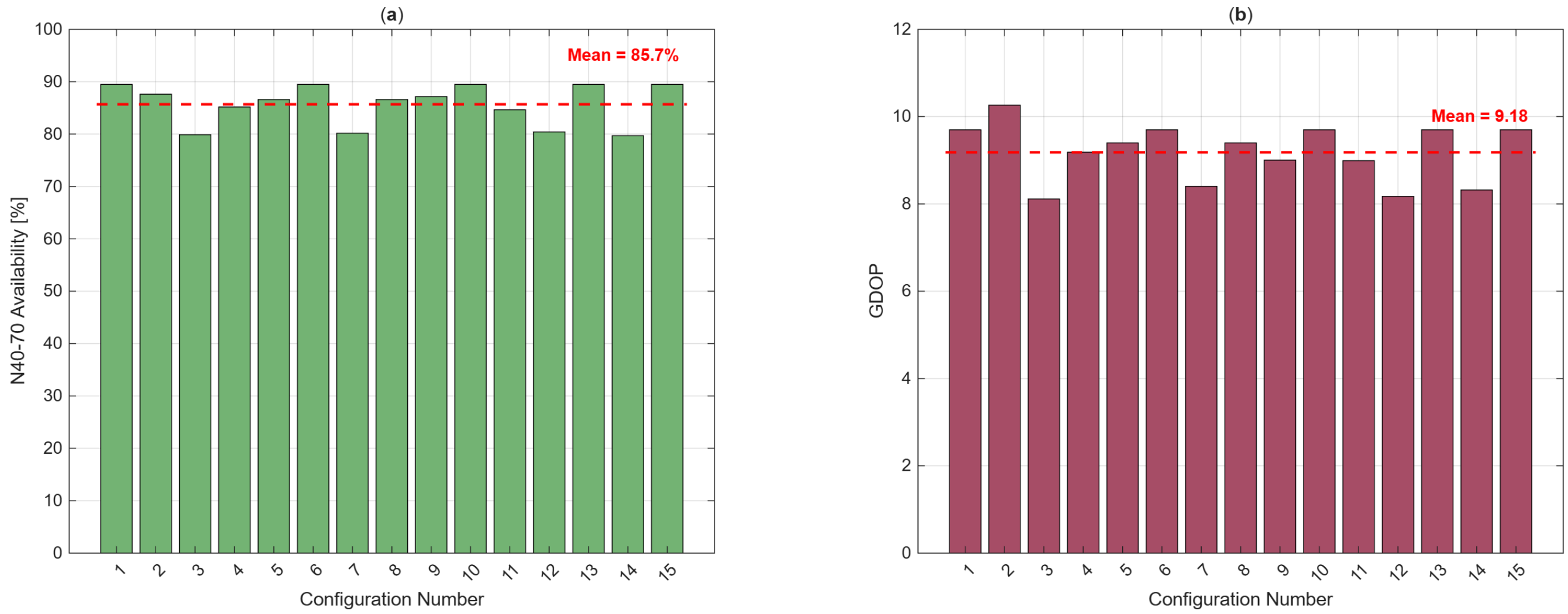

Figure S7. Weight sensitivity across 15 weight configurations: (**a**) N40–70 availability; (**b**) N40–70 GDOP. All 15 configurations exceed 79% availability, with the spread (9.8%p) narrower than observed in unfiltered formulations.

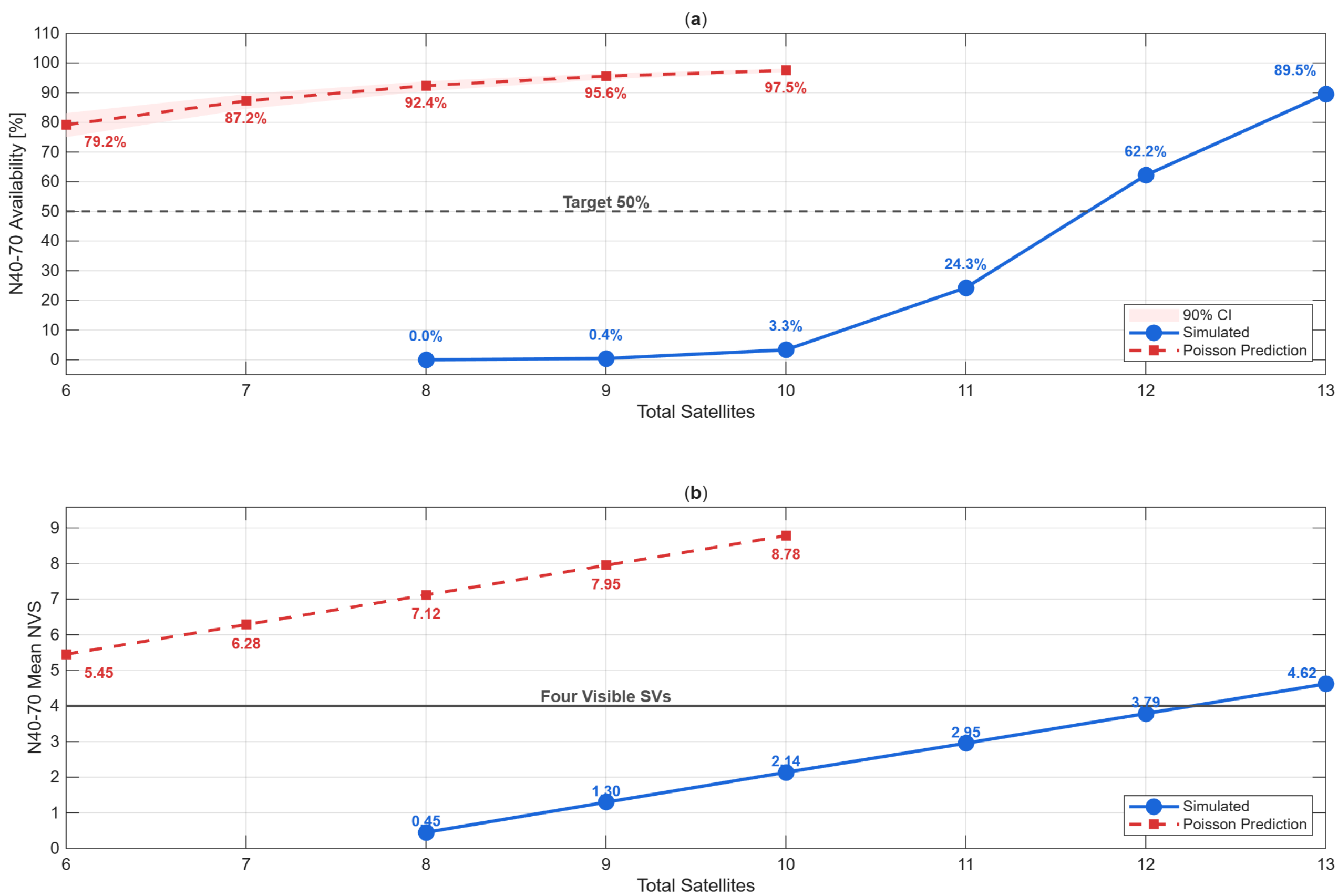

Figure S8. Scalability analysis vs. total satellite count, with Poisson prediction and 90% confidence interval: (**a**) PNT availability; (**b**) mean NVS. The simulated points (8–13 satellites) follow a sigmoidal growth, and the Poisson model projects ∼95% availability at 14–15 satellites.

Supplementary Tables

Table S1. Incremental PNT performance (168 h, perturbed model, $\varepsilon_{\min} = 5°$).

Metric	Base(8)	+1(9)	+2(10)	+3(11)	+4(12)	+5(13)
N-ROI NVS	0.45	1.30	2.14	2.95	3.79	4.62
N-ROI GDOP	—	9.91	8.09	7.85	9.79	9.70
N-ROI Avail [%]	0.00	0.40	3.30	24.30	62.20	89.50
S-ROI NVS	6.13	6.21	6.30	6.42	6.52	6.62
S-ROI GDOP	7.63	7.46	7.21	6.93	6.74	6.51
S-ROI Avail [%]	99.10	99.20	99.30	99.40	99.50	99.50
S. Pole Avail [%]	99.4	99.4	99.7	99.7	99.7	99.7
Global Avail [%]	42.1	45.2	49.6	59.7	75.9	86.9

Table S2. Keplerian vs. perturbed divergence over 720 hours.

Metric	Keplerian	Perturbed	Δ
N-ROI Avail [%]	87.66	89.05	+1.39
N-ROI GDOP	9.89	10.06	+0.17

Table S3. N-ROI availability at multiple GDOP thresholds (final 13-satellite constellation, 168 h, $\varepsilon_{\min} = 5°$).

GDOP Threshold	N-ROI Avail [%]	Reduction vs. GDOP < 50
< 50	89.5	—
< 30	87.9	−1.6%p
< 20	82.0	−7.5%p
< 15	70.5	−19.0%p

Table S4. Elevation mask sensitivity (user-side masking re-evaluation; the $\varepsilon_{\min} = 5°$ reference value does not coincide with the headline 89.5% of the Incremental Performance subsection owing to a discretisation difference between the two runs—the trend across mask values is the focus).

$\varepsilon_{\min}$	N-ROI NVS	N-ROI GDOP	N-ROI Avail [%]
5°	4.02	9.36	64.7
10°	3.71	11.30	53.8
15°	3.40	13.06	44.0

Table S5. PNT performance at KASA 2032 candidate landing sites (final 13-satellite constellation, 168 h, $\varepsilon_{\min} = 5°$).

Site	Lat.	Lon.	NVS	GDOP	Base Avail [%]	KLNS Avail [%]
Gärtner	+59.2°	+34.6°	4.70	9.30	0.0	92.5
Endymion	+53.6°	+56.5°	4.69	10.32	0.0	94.2
Lacus Mortis	+45.1°	+27.2°	4.76	9.35	0.0	94.2
Clavius	−58.6°	−14.4°	6.59	6.48	99.8	100.0
Pingré	−58.6°	−73.7°	6.72	5.48	99.6	99.7
Maginus	−50.0°	−6.2°	6.27	7.17	99.9	100.0

Table S6. S-band link budget analysis (2491.8 MHz). The "Altitude" column reports the satellite altitude above the lunar surface at the indicated orbital phase, used here as a first-order proxy for slant range under the simplifying assumption of a sub-satellite user. A more thorough budget would account for off-nadir geometry and would yield a larger slant range (and FSPL) at the limb of the visibility footprint.

**Scenario**	**Altitude [km]**	**FSPL [dB]**	C/N_0 **[dBHz]**
Apolune (high)	18,663	185.8	32.2
Mid-range	9,158	179.6	38.4
Perilune (low)	1,863	165.7	52.3

Table S7. Stability pre-filter outcome.

**Stage**	**Count**
Total candidate orbits ($a \times e \times i \times \Omega \times M_0$)	10,368
After perilune filter ($r_p - R_{\text{Moon}} > 100$ km)	10,368
Removed by stability filter (Eqs. (5)–(8) of main text)	2,864
**Final stable candidate pool**	**7,504**

Supplementary Notes

RK4 Step-Size Convergence Study

To justify the fixed 60-second integration step used throughout this study, we performed a step-size convergence analysis on the five selected KLNS satellites (Table 3 of the main text) over the full 720-hour validation horizon. Each trajectory was propagated with the perturbed dynamical model under step sizes $\Delta t \in \{240, 120, 60, 30, 15\}$ s, and the final state was compared against a fine reference solution computed with $\Delta t_{\text{ref}} = 7.5$ s.

Table S8 reports the worst-case (over the five satellites) final position error and osculating-element errors. At the adopted step of $\Delta t = 60$ s the maximum final position error after 720 hours is only 0.14 km, and the corresponding semi-major-axis error is 2.4×10^{-3} km—a relative error of 2.0×10^{-7}, roughly six orders of magnitude below the stability-filter threshold $\Delta a/a < 0.40$. The empirically observed convergence order, obtained from successive step halving, is $p \approx 4.7$–4.9, consistent with the fourth-order theoretical accuracy of the RK4 scheme and confirming that $\Delta t = 60$ s lies well within the asymptotic regime. The 60-second step therefore introduces numerical error that is negligible relative to the physical perturbation effects under study, while keeping the 10,368-candidate, 720-hour stability screen computationally tractable.

Table S8. RK4 step-size convergence over the 720-hour horizon for the five KLNS satellites. Errors are worst-case over KLNS-1–5, evaluated against a $\Delta t_{\text{ref}} = 7.5$ s reference solution. The empirical order p is obtained from successive halving of the mean position error.

Δt (s)	Max position error (km)	Max $\lvert\Delta a\rvert$ (km)	Max $\lvert\Delta i\rvert$ (deg)	Order p
240	1.28×10^{2}	2.35×10^{0}	3.10×10^{-3}	—
120	4.20×10^{0}	7.47×10^{-2}	1.68×10^{-4}	4.93
60	1.42×10^{-1}	2.39×10^{-3}	9.66×10^{-6}	4.88
30	5.11×10^{-3}	7.78×10^{-5}	6.41×10^{-7}	4.78
15	1.94×10^{-4}	2.54×10^{-6}	4.13×10^{-8}	4.70

Validation Against a Degree/Order-16 Lunar Gravity Field

To assess how the results change under a higher-fidelity gravity model, we re-propagated the five selected KLNS satellites over the 720-hour horizon under the GSFC GRGM1200A lunar gravity field truncated to degree and order 16, and compared the resulting orbital evolution against the J_2-only model used in the main analysis. The degree/order-16 field is evaluated in the lunar principal-axes body-fixed frame, which rotates at the sidereal rate ($2\pi/27.32$ day^{-1}); at each RK4 step the satellite position is rotated into this frame, the spherical-harmonic acceleration is computed via the Cunningham recursion, and the result is rotated back to the inertial frame. Both models use the same gravitational parameter and reference radius, so the comparison isolates the higher-order and tesseral gravitational contributions. The implementation was verified to reproduce the analytic point-mass and J_2 accelerations to machine precision.

Table S9 compares the two models. The four stability-filter quantities—maximum eccentricity $e_{\max}$, relative semi-major-axis range $\Delta a/a$, inclination range Δi, and minimum periapsis radius $r_{p,\min}$—are essentially identical under the two models (relative changes below 1%), and all five satellites continue to satisfy the four stability criteria under the degree/order-16 field. The final-position divergence between the two models after 720 hours ranges from 1.7 to 81 km, small relative to the $\sim$12,000 km orbital length scale. Although the higher-order gravitational acceleration is up to $\sim$30 times larger near periapsis ($r_p \approx 3{,}600$ km) than near apoapsis, its cumulative effect over the 30-day window does not alter the stability classification of any selected orbit. This confirms that the J_2-only model adopted in the main analysis is adequate for the long-horizon stability assessment of the selected high-altitude ($a = 12{,}000$ km, $e = 0.70$) constellation.

Table S9. Comparison of 720-hour orbital evolution under the J_2-only and degree/order-16 (d/o16) lunar gravity models for the five selected KLNS satellites. Δr_{final} is the final-position difference between the two models after 720 hours. All five satellites pass the four stability-filter criteria under both models.

Sat	Model	$e_{\max}$	$\Delta a/a$	Δi (deg)	$r_{p,\min} - R_M$ (km)
KLNS-1	J_2	0.7762	5.89×10^{-3}	3.237	949.6
	d/o16	0.7762	5.88×10^{-3}	3.236	949.5
KLNS-2	J_2	0.7727	6.49×10^{-3}	5.516	1006.3
	d/o16	0.7727	6.49×10^{-3}	5.516	1006.2
KLNS-3	J_2	0.7752	6.04×10^{-3}	4.262	962.0
	d/o16	0.7752	6.03×10^{-3}	4.259	961.9
KLNS-4	J_2	0.7026	6.49×10^{-3}	3.272	1851.2
	d/o16	0.7026	6.52×10^{-3}	3.274	1851.3
KLNS-5	J_2	0.7782	5.70×10^{-3}	5.060	924.5
	d/o16	0.7783	5.70×10^{-3}	5.065	924.4

Final-position divergence Δr_{final} (J_2 vs d/o16, 720 h): KLNS-1 2.10, KLNS-2 2.74, KLNS-3 1.68, KLNS-4 3.56, KLNS-5 81.16 km.
